# Zinc-limited *Mycobacterium tuberculosis* stimulate distinct responses in macrophages compared with standard zinc-replete bacteria

**DOI:** 10.1128/iai.00578-24

**Published:** 2025-02-04

**Authors:** Endrei Marcantonio, Allexa D. Burger, Kelly H. Chang, Fukun W. Hoffmann, Yuanyuan Fu, Vedbar S. Khadka, Benoit J. Smagghe, Youping Deng, Peter R. Hoffmann, Sladjana Prisic

**Affiliations:** 1 School of Life Sciences, University of Hawai'i at Mānoa 3949 , Honolulu, Hawaii, USA; 2 Department of Cell and Molecular Biology, John A. Burns School of Medicine, University of Hawai'i at Mānoa 231410 , Honolulu, Hawaii, USA; 3 Department of Quantitative Health Sciences, John A. Burns School of Medicine, University of Hawai'i at Mānoa 3949 , Honolulu, Hawaii, USA; University of Pennsylvania Perelman School of Medicine, Philadelphia, Pennsylvania, USA

**Keywords:** *Mycobacterium tuberculosis*, macrophages, zinc

## Abstract

Tuberculosis (TB) is notoriously difficult to treat, likely due to the complex host-pathogen interactions driven by pathogen heterogeneity. An understudied area of TB pathogenesis is host responses to *Mycobacterium tuberculosis* bacteria (Mtb) that are limited in zinc ions. This distinct population resides in necrotic granulomas and sputum and could be the key player in tuberculosis pathogenicity. In this study, we tested the hypothesis that macrophages differentiate between Mtb grown under zinc limitation or in the standard zinc-replete medium. Using several macrophage infection models, such as murine RAW 264.7 and murine bone marrow-derived macrophages (BMDMs), as well as human THP-1-derived macrophages, we show that macrophages infected with zinc-limited Mtb have increased bacterial burden compared with macrophages infected with zinc-replete Mtb. We further demonstrate that macrophage infection with zinc-limited Mtb trigger higher production of reactive oxygen species (ROS) and cause more macrophage death. Furthermore, the increased ROS production is linked to the increased phagocytosis of zinc-limited Mtb, whereas cell death is not. Finally, transcriptional analysis of RAW 264.7 macrophages demonstrates that macrophages have more robust pro-inflammatory responses when infected with zinc-limited Mtb than zinc-replete Mtb. Together, our findings suggest that Mtb’s access to zinc affects their interaction with macrophages and that zinc-limited Mtb may be influencing TB progression. Therefore, zinc availability in bacterial growth medium should be considered in TB drug and vaccine developments.

## INTRODUCTION

*Mycobacterium tuberculosis* bacteria (Mtb) cause tuberculosis (TB), one of the deadliest human infectious diseases. With 1.25 million people killed by TB in 2023 and an estimated one-quarter of the world’s population latently infected, TB remains a significant public health issue (1). There are no effective vaccines for TB, antibiotic therapy is long and complex (2), and drug resistance is becoming more prevalent in some parts of the world (1), thus highlighting the need for novel TB vaccines and therapies. One factor contributing to TB’s challenging treatment is the heterogeneity of Mtb, that is, phenotypically distinct populations of Mtb *in vivo* (3). However, it is mostly unknown how the host immune system responds to phenotypically heterogeneous populations of Mtb. Considering that the host immune cells have key roles in protection against the infection and disease progression (4), it is reasonable to expect that phenotypically heterogeneous populations of Mtb can differentially modulate the host immune cell responses and that this modulation could have important implications in Mtb pathogenesis.

Mtb encounter different microenvironments within the host. Initially, Mtb infect new hosts via aerosols, which are inhaled into the lungs and phagocytized by alveolar macrophages. Immune cells recruited at the site of infection form complex cellular structures called granulomas (4). Granulomas often become necrotic, the hallmark of active TB (4). The formation of necrotic granulomas is mainly driven by neutrophils, which infiltrate solid granulomas and die. Many infected macrophages also die, thereby releasing Mtb into the necrotic granuloma’s extracellular milieu (5). Hence, Mtb can occupy two distinct microenvironments: (i) intracellular, that is, within cells such as macrophages, and (ii) extracellular, that is, within the necrotic core of granulomas (4). Mtb residing intracellularly and extracellularly encounter different signals and stresses, which may trigger the formation of distinct bacterial populations in these microenvironments.

One of the most striking differences between intracellular and extracellular environments in necrotic granulomas is zinc ion (Zn^2+^) availability. Intracellular Mtb encounter high concentrations of Zn^2+^ due to the accumulation of Zn^2+^ in Mtb-infected phagosomes (6). In contrast, extracellular Mtb encounter limiting availability of Zn^2+^ due to the accumulation of the neutrophil-derived Zn^2+^-chelating protein, calprotectin, in the necrotic regions of granulomas (7, 8). Therefore, this difference in Zn^2+^ availability triggers the formation of two distinct populations of Mtb in necrotic granulomas: (i) Zn^2+^-replete Mtb and (ii) Zn^2+^-limited Mtb (8, 9). We previously demonstrated that Mtb differentially express genes and remodel their proteomes and lipidomes when grown in Zn^2+^-limited medium vs. the standard Zn^2+^-replete medium (8). We noted additional differences between these two bacterial populations: Zn^2+^-limited Mtb have unique cell wall fibrils that Zn^2+^-replete Mtb lack, have different susceptibilities to certain antibiotics, have increased resistance to oxidative stress, and are more virulent than Zn^2+^-replete Mtb in the C3HeB/FeJ mouse infection model (8). Despite the relevance of Zn^2+^ limitation in TB pathogenesis (8, 10, 11), knowledge about this unique bacterial population and, more broadly, the effects of Mtb physiology caused by Zn^2+^ limitation on host responses is scarce.

Given the remarkable differences in the physiology between Zn^2+^-limited Mtb and Zn^2+^-replete Mtb, we sought to determine the effects of Zn^2+^-limited Mtb on macrophage responses. To understand how macrophages interact with these two distinct Mtb populations, we performed functional assays and demonstrated that Zn^2+^-limited Mtb cause higher bacterial burden, increased production of reactive oxygen species (ROS), and more macrophage death than Zn^2+^-replete Mtb. In addition, we performed transcriptomics on macrophages infected with Zn^2+^-limited Mtb and Zn^2+^-replete Mtb. We found that macrophages infected with Zn^2+^-limited Mtb more robustly upregulate inflammatory responses when compared with macrophages infected with Zn^2+^-replete Mtb. These data provide important insights into how macrophages respond to Zn^2+^ limited Mtb and further support the need to investigate the effects of Zn^2+^ limitation and phenotypic heterogeneity on host responses.

## RESULTS

### Zn^2+^-limited Mtb cause higher bacterial burden in macrophages

In our previous study, we used the pathogenic Mtb H37Rv (Mtb-Rv) and non-pathogenic Mtb mc^2^6206 (Mtb-Aux) strains to demonstrate that dramatic physiological changes occur in response to Zn^2+^ limitation in both strains (8). Here, we continued using the Mtb-Aux to study their interactions with macrophages because (i) Mtb-Aux can be safely handled in a BSL2 laboratory, and (ii) it should elicit similar macrophage responses as Mtb-Rv (12). We also exclusively look at the early steps of infection. We have previously demonstrated that Mtb grown in Zn^2+^-limited medium (Mtb-ZLM) have altered lipidome and distinct cell surface morphology vs. Mtb grown in Zn^2+^-replete medium (Mtb-ZRM) (8). Since the lipidous cell wall is vital for Mtb interactions with macrophages (13) and their interactions have a critical role in the outcome of infection (14), we did functional assays to determine whether bacterial uptake was different between Mtb-Aux grown in Zn^2+^-replete medium (Aux-ZRM) and Mtb-Aux grown in Zn^2+^-limited medium (Aux-ZLM).

**Fig 1:**
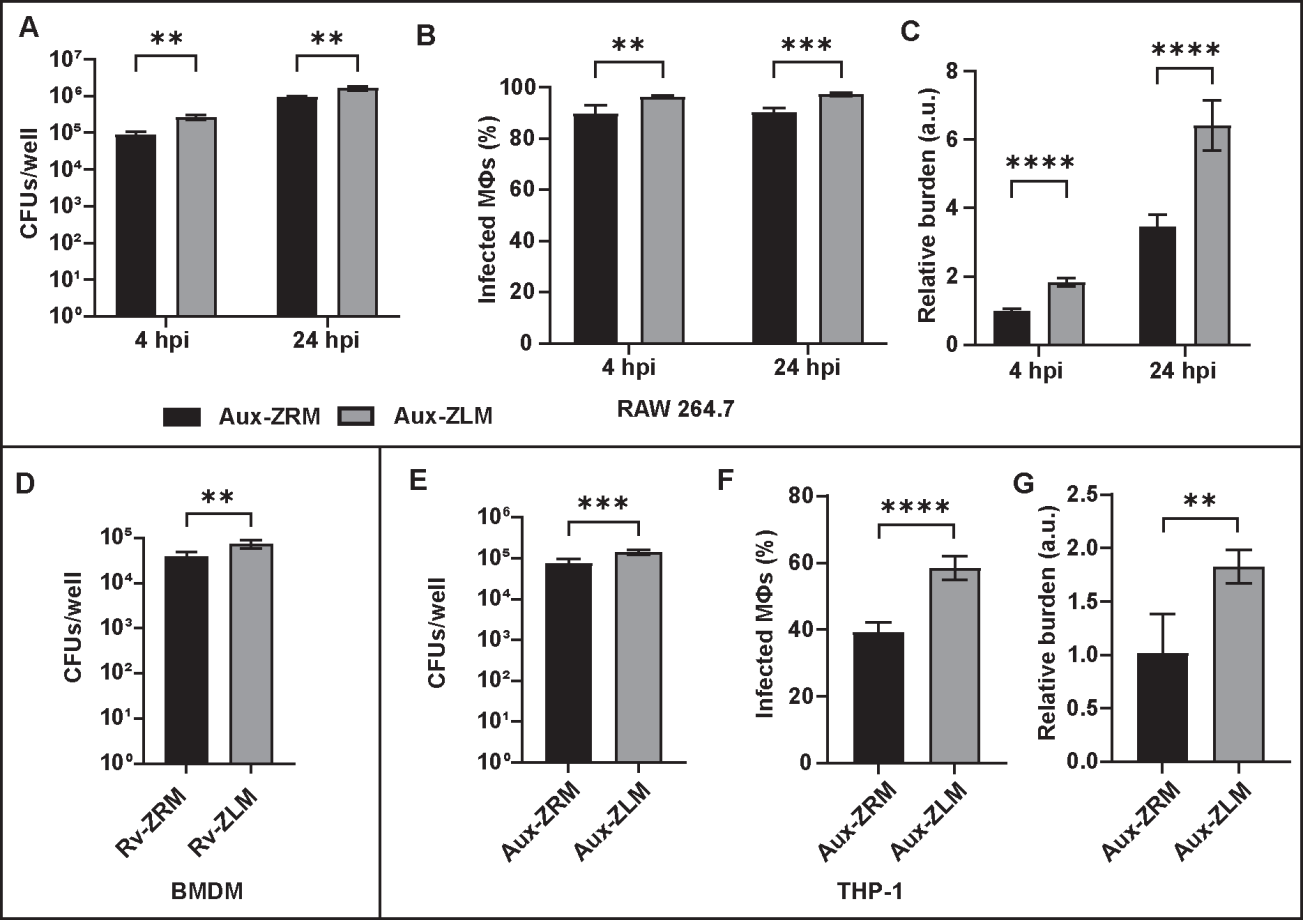
Macrophages more readily phagocytose Zn^2+^-limited Mtb than Zn^2+^-replete Mtb. (A) Intracellular CFUs of Aux-ZRM and Aux-ZLM in RAW 264.7 cells at 4 and 24 hpi. (B) The percentage and (C) relative bacterial burden of RAW 264.7 macrophages infected with DsRed-expressing Aux-ZRM and Aux-ZLM at 4 and 24 hpi, as determined by flow cytometry. (D) Intracellular CFUs of Rv-ZRM and Rv-ZLM in murine BMDMs at 4 hpi. (E) Intracellular CFUs of Aux-ZRM and Aux-ZLM in THP-1 macrophages at 4 hpi. (F) The percentage and (G) relative bacterial burden of THP-1 macrophages infected with GFP-expressing Aux-ZRM and Aux-ZLM at 2 hpi, as determined by flow cytometry. Bacterial burden is relative to the burden of macrophages infected with Aux-ZRM. The error bars are the standard deviations of six replicates. Representative data from at least two independent experiments are shown. **: *P*-value < 0.01. ***: *P*-value < 0.001. ****: *P*-value < 0.0001. CFUs: colony forming units; Aux-ZRM and Aux-ZLM: Mtb mc^2^6206 grown in Zn^2+^-replete or limited media, respectively; Rv-ZRM and Rv-ZLM: Mtb H37Rv grown in Zn^2+^-replete or limited media, respectively; BMDM: bone marrow-derived macrophages. MΦ: macrophage. a.u.: arbitrary units.

Murine RAW 264.7 macrophages were infected with Aux-ZRM and Aux-ZLM at a multiplicity of infection of 20 (MOI 20), and the uptake of Mtb was determined at 4 h post-infection (hpi) and 24 hpi by counting the intracellular colony-forming units (CFUs). There were small, but statistically significant, increases in CFUs of Aux-ZLM vs. Aux-ZRM at 4 hpi and 24 hpi, indicating that there was an increased uptake (i.e., phagocytosis) of Aux-ZLM (Fig. 1A). We sought to determine if the increased uptake of Aux-ZLM was due to increased infectivity (i.e., more infected macrophages), increased bacterial burden (i.e., higher intracellular load per macrophage), or a combination of these two factors. To determine the cause of the increased uptake of Aux-ZLM, macrophages were infected with DsRed-expressing fluorescent Aux-ZRM and Aux-ZLM and analyzed at 4 and 24 hpi by flow cytometry. The percentages of infected macrophages and their bacterial burden were compared in macrophages infected with Aux-ZLM and macrophages infected with Aux-ZRM. Almost all macrophages were infected at 4 hpi, but there was a small increase in the percentage of macrophages infected with fluorescent Aux-ZLM vs. Aux-ZRM, and this trend remained at 24 hpi (Fig. 1B). In contrast, there were marked increases in the median fluorescence intensity (i.e., bacterial burden) in macrophages infected with Aux-ZLM at both 4 and 24 hpi, compared with Aux-ZRM (Fig. 1C). There was no difference in DsRed fluorescence intensity in cultures of Aux-ZLM vs. Aux-ZRM (Fig. S1A), suggesting that the increased fluorescent signal was due to increased bacterial burden rather than differences in fluorescent intensity of Aux-ZLM vs. Aux-ZRM strains. To determine if the increased burden was due to increased intracellular Aux-ZLM vs. Aux-ZRM or increased adhesion of Aux-ZLM vs. Aux-ZRM to macrophages (i.e., extracellular bacteria adhering to macrophage membranes), macrophages were infected with fluorescent Aux-ZRM and Aux-ZLM, and the extracellular bacteria were removed by extensive washing and visualized using confocal microscopy at both 4 and 24 hpi time points. There were qualitative increases in intracellular Aux-ZLM vs. Aux-ZRM at both 4 hpi and 24 hpi (Fig. S2A and B; Videos S1 and S2), suggesting that the increased burden was indeed due to increased intracellular Aux-ZLM vs. Aux-ZRM in macrophages rather than increased adhesion. In summary, RAW 264.7 macrophages phagocytosed more Aux-ZLM vs. Aux-ZRM, and the increased uptake was mainly due to the increased bacterial burden rather than different infectivities.

The RAW 264.7 cell line is immortalized and may have aneuploidy and/or other genetic modifications that could affect their functions (15, 16). To determine if the increased phagocytosis was due to extraneous effects related to cell line immortalization or also occurred in primary cells, bone marrow-derived macrophages (BMDMs) were prepared from C57BL/6 mice. In addition, although we previously demonstrated that attenuated Mtb-Aux and virulent Mtb-Rv have similar phenotypes *in vitro* when grown in ZRM and ZLM (8), we also sought to validate our findings with Mtb-Rv. BMDMs were infected with Mtb-Rv grown in ZRM (Rv-ZRM) and Mtb-Rv grown in ZLM (Rv-ZLM) at MOI 10 to determine uptake. As expected, there was a small but significant increase in intracellular Rv-ZLM vs. Rv-ZRM at 4 hpi (Fig. 1D). Therefore, there was increased phagocytosis of Mtb-ZLM vs. Mtb-ZRM by both the RAW 264.7 cell line and murine BMDMs.

To determine if there was increased phagocytosis by human cells, we differentiated the THP-1 cell line into macrophage-like cells and infected them with Aux-ZRM and Aux-ZLM at MOI 10 and measured intracellular burden at 4 hpi. There was a significant increase in CFUs obtained from THP-1 macrophages infected with Aux-ZLM vs. Aux-ZRM (Fig. 1E). After confirming that GFP fluorescence did not change when grown in ZRM and ZLM (Fig. S1B), we infected THP-1 macrophages with GFP-expressing Aux-ZRM and Aux-ZLM and measured the uptake of bacteria at 2 hpi. There were increases in the percentage of infected macrophages and bacterial burden of macrophages infected with Aux-ZLM vs. Aux-ZRM (Fig. 1F and G). In summary, the phagocytosis phenotype is consistent between various macrophage infection models that are tested, thus showing that Zn^2+^-limited Mtb infect more macrophages and cause greater bacterial burden than Mtb grown in standard Zn^2+^-replete medium.

### Macrophages have increased production of reactive oxygen species when infected with Zn^2+^-limited Mtb

**Fig 2:**
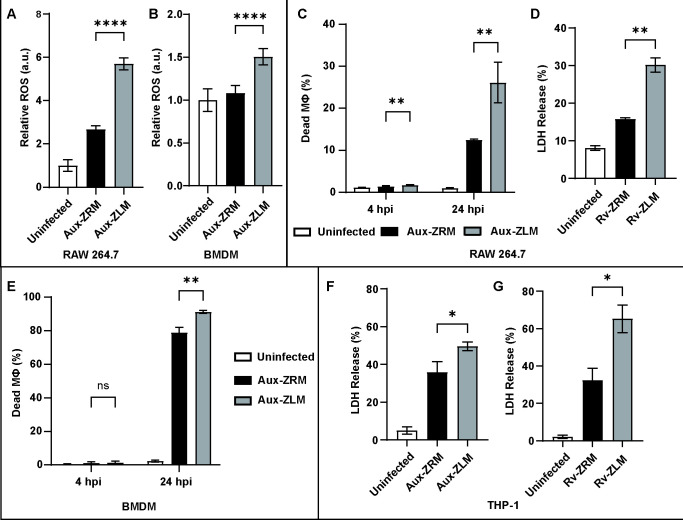
Macrophages increase the production of ROS and die more when infected with Zn^2+^-limited Mtb. Production of ROS in (A) RAW 264.7 macrophages and (B) BMDMs infected with Aux-ZRM and Aux-ZLM at 4 hpi. The production of ROS was relative to uninfected macrophages. (C) Percentage of dead RAW 264.7 macrophages when infected with Aux-ZRM and Aux-ZLM at 4 and 24 hpi, as determined by flow cytometry. (D) LDH release by uninfected RAW 264.7 macrophages or infected with Rv-ZRM or Rv-ZLM at 24 hpi. (E) Percentage of dead BMDMs infected with Aux-ZRM and Aux-ZLM at 4 and 24 hpi, as determined by flow cytometry.(F) LDH is released by uninfected THP-1 macrophages or infected with Aux-ZRM or Aux-ZLM at 24 hpi. (G) LDH is released by uninfected THP-1 macrophages or infected with Rv-ZRM or Rv-ZLM at 24 hpi. The LDH release was relative to the total LDH released by lysed RAW 264.7 or THP-1 macrophages. The error bars are the standard deviations of six replicates. The data shown are from a representative assay of at least two independent experiments. *: *P*-value < 0.05. **: *P*-value < 0.01. ****: *P*-value < 0.0001. ROS: reactive oxygen species; LDH: lactate dehydrogenase. See Fig. 1 for other abbreviations.

The increased phagocytosis in macrophages infected with Aux-ZLM vs. Aux-ZRM could indicate that other macrophage functions, such as ROS production, differ when infected with these two Mtb populations. Macrophages produce large amounts of ROS to eradicate pathogens during phagocytosis. NADPH oxidase is assembled on phagosomes in response to the recognition of microbial-associated molecular patterns by pattern recognition receptors, such as Toll-like receptors (17). Given that there was an increased uptake of Aux-ZLM, we hypothesized that there would be increased production of ROS in macrophages infected with Aux-ZLM vs. Aux-ZRM. To test this hypothesis, RAW 264.7 macrophages were infected with Aux-ZRM and Aux-ZLM, and ROS production was evaluated at 4 hpi using flow cytometry. As hypothesized, there was a marked increase in ROS in macrophages infected with Aux-ZLM vs. Aux-ZRM (Fig. 2A). We performed a validation assay using BMDMs that were infected with Aux-ZRM and Aux-ZLM. (Note that the ROS assay in THP-1 macrophages was not included here because we observed that these cells do not produce ROS at early time points during the infection, which was reported previously (18), and measurements at later time points were complicated by the differences in cell death, as shown below.) ROS production in BMDMs was measured at 4 hpi, and it was found that BMDMs infected with Aux-ZLM had elevated levels of ROS vs. BMDMs infected with Aux-ZRM (Fig. 2B). Thus, macrophages infected with Aux-ZLM produce higher amounts of ROS than macrophages infected with Aux-ZRM.

### Zn^2+^-limited Mtb induce more macrophage death

Host cell death has an integral role in host-pathogen interactions, and Mtb can induce host cell death using a variety of mechanisms (19). It is known that a high bacterial burden of Mtb in macrophages induce necrotic cell death (20–22). Since we observed there was increased bacterial burden and ROS in macrophages infected with Aux-ZLM vs. macrophages infected with Aux-ZRM, we investigated if there were differences in macrophage death when infected with Aux-ZLM vs. Aux-ZRM at 4 and 24 hpi by flow cytometry. There was a marginal increase in macrophage death at 4 hpi when macrophages were infected with Aux-ZLM vs. Aux-ZRM (Fig. 2C), and a striking increase in macrophage death at 24 hpi when macrophages were infected with Aux-ZLM vs. Aux-ZRM (Fig. 2C). These results suggested that Zn^2+^-limited Mtb induce more macrophage death than Zn^2+^-replete Mtb.

We did validation assays with Mtb-Rv to determine if the virulent strain triggered increased cell death as well. We were unable to use flow cytometry with the virulent strain due to biosafety constraints; hence, we determined cell death of RAW 264.7 macrophages at 24 hpi using lactate dehydrogenase (LDH) release assays. As expected, there was a significant increase of LDH release in RAW 264.7 macrophages infected with Rv-ZLM vs. Rv-ZRM, comparable with the difference observed for Aux-ZLM vs. Aux-ZRM (Fig. 2D). Furthermore, we validated this phenotype in BMDMs using Mtb-Aux and flow cytometry. There was no difference in macrophage death at 4 hpi and an increase in macrophage death at 24 hpi (Fig. 2E). Finally, we investigated if Aux-ZLM and Rv-ZLM triggered increased cell death compared with Aux-ZRM and Rv-ZRM in human macrophages using THP-1 macrophages. THP-1 macrophages were infected with Mtb-Aux and Mtb-Rv, and cell death was determined at 24 hpi. There were marked increases in LDH release by THP-1 macrophages infected with Aux-ZLM vs. Aux-ZRM and Rv-ZLM vs. Rv-ZRM (Fig. 2F and G). In summary, there was more pronounced macrophage death caused by both attenuated and virulent Mtb-ZLM vs. Mtb-ZRM, which occurred in both cell lines and primary cells.

### Effects of phagocytosis inhibition on production of ROS and macrophage death

Aux-ZLM triggered increased ROS production by RAW 264.7 macrophages compared with Aux-ZRM. It was hypothesized that this response is due to the increased uptake of Aux-ZLM vs. Aux-ZRM. To test this hypothesis, the effect of phagocytosis inhibition on ROS production was assessed. RAW 264.7 macrophages were treated with 3 µM Cytochalasin D (CytoD), a phagocytosis inhibitor, prior to infection, to partially inhibit and equalize phagocytosis, and macrophages were infected with fluorescent Aux-ZRM and Aux-ZLM at MOI 20.

**Fig 3:**
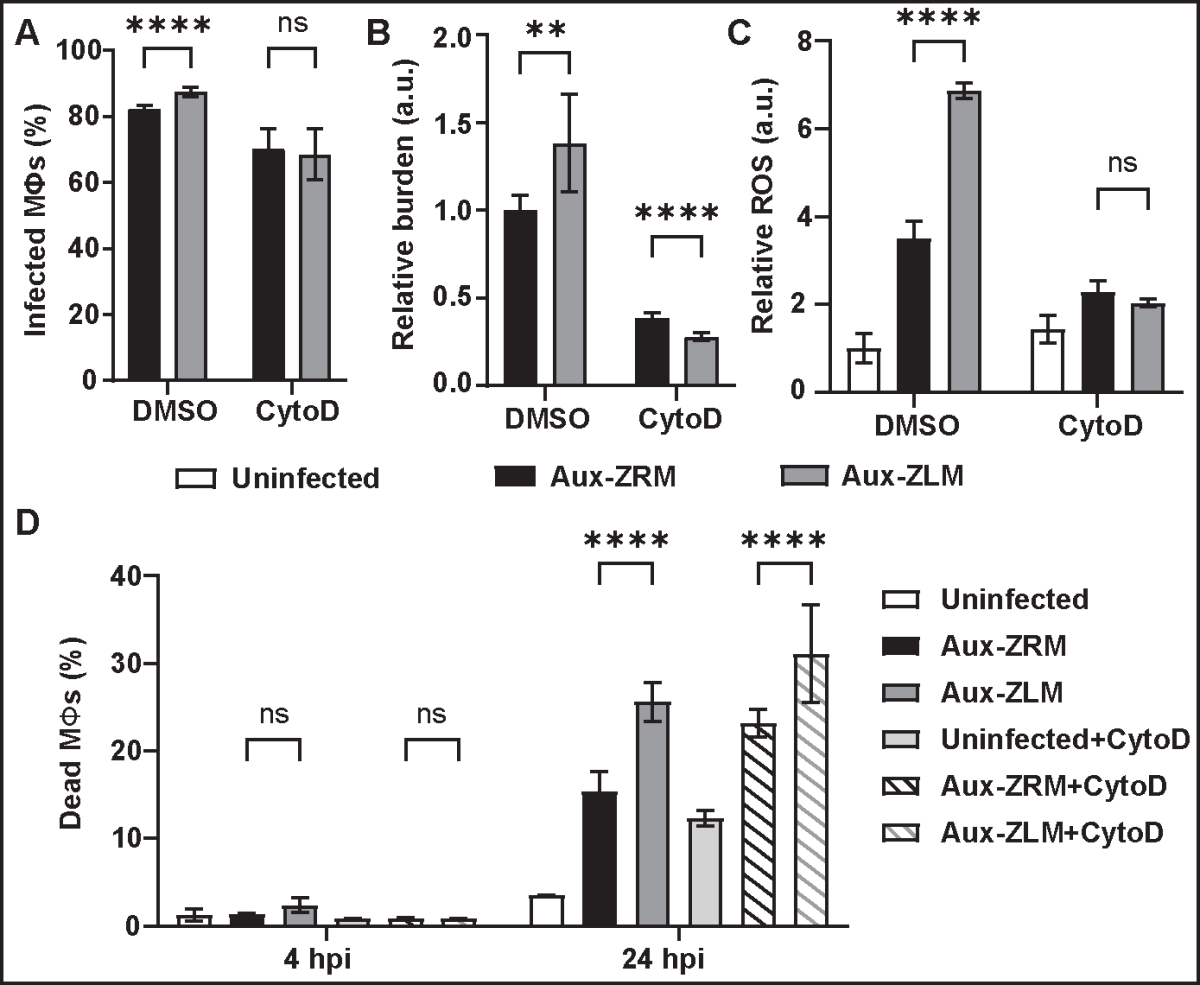
The effect of phagocytosis inhibition on ROS production and cell death in RAW 264.7 macrophages. (A) Percentage of infected macrophages, (B) relative bacterial burden, and (C) production of ROS in macrophages infected with GFP-expressing Aux-ZRM and Aux-ZLM in the presence or absence of phagocytosis inhibitor (CytoD). (D) Percentage of uninfected and infected dead macrophages that were untreated or treated with CytoD, as determined by flow cytometry. The error bars are the standard deviations of six replicates. The data shown are from a representative assay of at least two independent experiments. **: *P*-value < 0.01. ****: *P*-value < 0.0001. CytoD: cytochalasin D; ns: not significant. See Fig. 1 for other abbreviations.

As expected, there was a small increase in the percentage of infected macrophages when untreated control macrophages were infected with Aux-ZLM vs. Aux-ZRM, whereas CytoD treatment reduced infection and abolished the difference between Aux-ZRM and Aux-ZLM (Fig. 3A). Once again, the untreated control showed increased bacterial burden when infected with Aux-ZLM vs. Aux-ZRM in the presence of DMSO (Fig. 3B), whereas the bacterial burden was greatly diminished in CytoD-treated macrophages, and curiously, there was a small, but significant decrease in bacterial burden of Aux-ZLM vs. Aux-ZRM (Fig. 3B). Regarding the production of ROS, there was a significant increase in ROS in untreated control macrophages infected with Aux-ZLM vs. Aux-ZRM, and no difference in CytoD-treated infected macrophages (Fig. 3C). Altogether, the disruption of formation of actin filaments with CytoD may affect phagocytosis of Aux-ZLM aggregates more prominently in comparison to Aux-ZRM. Importantly, the increase in ROS production depended on phagocytosis and was driven by the higher bacterial burden seen with Aux-ZLM.

The bacterial burden of Mtb is known to be a decisive factor in host cell death, as the high bacterial burden of Mtb and aggregated Mtb can induce rapid host cell death (21, 22). However, it has previously been shown that extracellular aggregates of Mtb can also induce rapid cell death in the absence of phagocytosis (23). To test if the increased macrophage death with Aux-ZLM was dependent on bacterial burden (i.e., uptake), RAW 264.7 macrophages were treated with CytoD and infected with Aux-ZRM and Aux-ZLM. Macrophage death was determined at 4 and 24 hpi by flow cytometry. There were no differences in dead macrophages with Aux-ZLM vs. Aux-ZRM at 4 hpi, regardless of phagocytosis inhibition (Fig. 3D). However, there were significant increases in dead macrophages with Aux-ZLM vs. Aux-ZRM at 24 hpi in both untreated and CytoD-treated macrophages (Fig. 3D). There was also an increase in macrophage death when macrophages were treated with CytoD for 24 h (Fig. 3D), indicating that long exposure to CytoD could be cytotoxic to macrophages. Regardless, the difference between Aux-ZLM and Aux-ZRM in causing macrophage death remained even when phagocytosis was inhibited (Fig. 3D). Therefore, in contrast to the ROS production, cell death of macrophages infected with Aux-ZLM may not be directly correlated to their increased bacterial burden, but due to the distinct interactions with extracellular bacteria.

### Characterization of macrophage transcriptional responses to Aux-ZRM and Aux-ZLM

We hypothesized that macrophages would have altered transcriptional responses to Mtb-ZLM compared with Mtb-ZRM due to the distinct physiology of Mtb-ZLM vs. Mtb-ZRM (8), as well as the differences in uptake, production of ROS, and cell death. Here, we used Mtb-Aux to study the macrophage responses by transcriptomics. The early macrophage responses were analyzed because Mtb-containing phagosomes accumulate Zn^2+^ within 24 h (6); hence, Zn^2+^-limited Mtb should quickly become Zn^2+^-replete after phagocytosis.

To determine how macrophages respond to Mtb-ZRM and Mtb-ZLM, RAW 264.7 macrophages were infected with Aux-ZRM and Aux-ZLM and were exposed to the bacteria for 4 h and 24 h, that is, extracellular bacteria were not removed at any time point, to account for *in vivo*-relevant signals from both intra- and extra-cellular Mtb (Fig. S3A). Aux-ZRM and Aux-ZLM induce largely similar macrophage responses, albeit with small differences in clustering between macrophages infected with Aux-ZRM and Aux-ZLM at 4 and 24 hpi (Fig. S3B). Differentially expressed genes (DEGs) were determined in the following comparisons: uninfected macrophages with ZLM vs. uninfected macrophages with ZRM at 4 h and 24 h, macrophages infected with Aux-ZRM vs. uninfected macrophages at 4 and 24 hpi, and macrophages infected with Aux-ZLM vs. uninfected macrophages at 4 and 24 hpi. These analyses showed that Zn^2+^ supplementation of bacterial growth media alone did not significantly affect gene expression in macrophages (Table S1), whereas infecting macrophages with Aux-ZRM or Aux-ZLM caused transcriptional changes for hundreds of genes (Table S2 through S5). Ingenuity Pathway Analysis (IPA), Kyoto Encyclopedia of Genes and Genomes (KEGG), and Gene Ontology (GO) pathway enrichment analyses were done, and they demonstrated that Aux-ZRM and Aux-ZLM induced highly similar pro-inflammatory responses at both 4 and 24 hpi (Table S6 through S13; Fig. S3C through F). Therefore, although Aux-ZRM and Aux-ZLM triggered similar macrophage responses, the small differences in clustering between macrophages infected with Aux-ZRM and Aux-ZLM at both time points prompted further investigation to determine whether there was differential modulation of macrophage responses by Aux-ZLM vs. Aux-ZRM.

### Zn^2+^-limited Mtb trigger more robust inflammatory responses in macrophages

**Fig 4:**
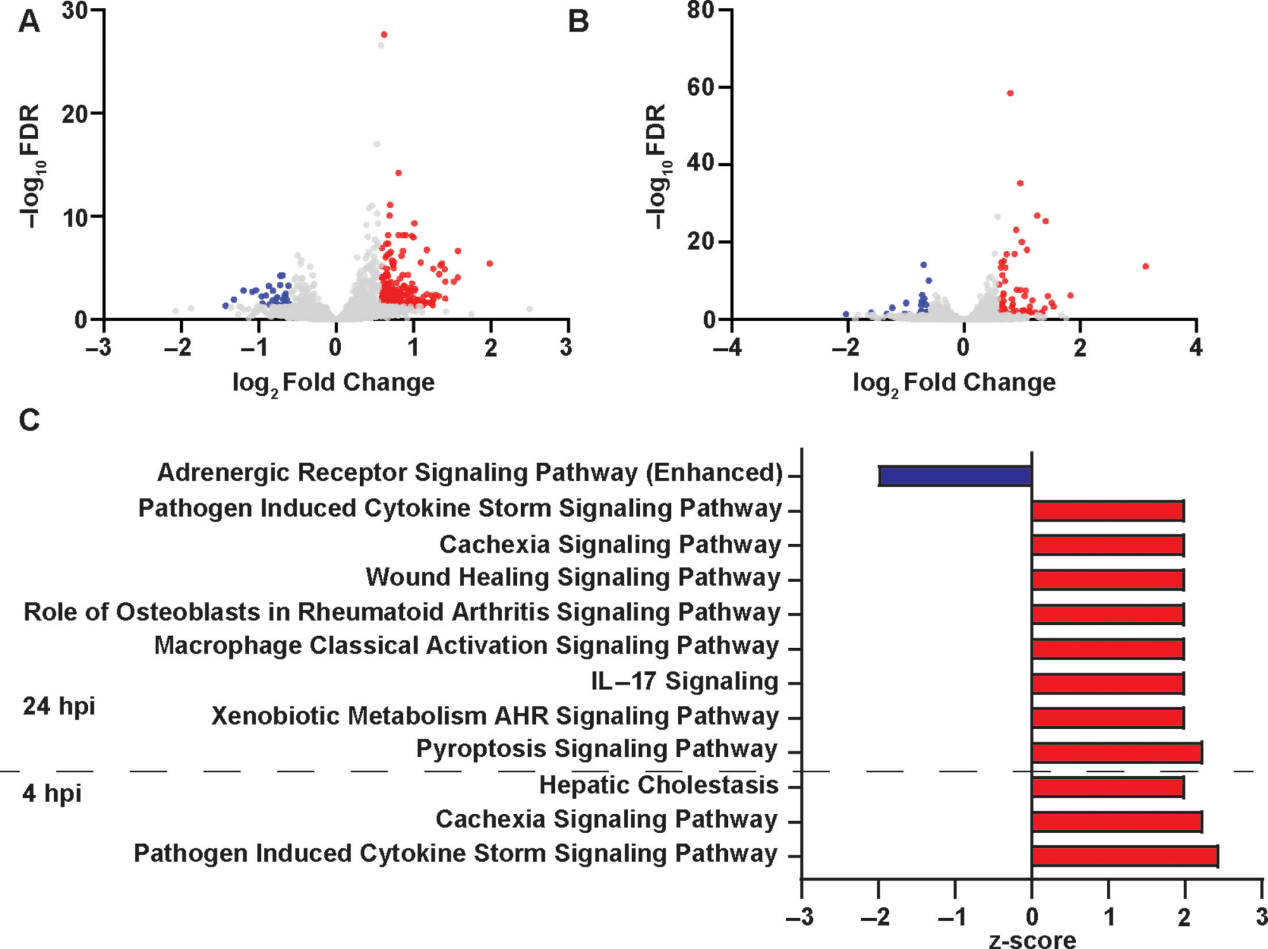
Aux-ZLM elicit increased inflammatory responses in RAW 264.7 macrophages compared with Aux-ZRM. (A) Volcano plot of DEGs in macrophages infected with Aux-ZLM vs. macrophages infected with Aux-ZRM at 4 hpi or (B) 24 h. (C) The top activated and inhibited pathways in macrophages infected with Aux-ZLM vs. macrophages infected with Aux-ZRM at 4 and 24 hpi. The pathways with *P*-value < 0.05 and z-score >2 or <-2 were considered significant. Blue indicates downregulation, and red indicates upregulation of genes or pathways. FDR: false discovery rate. DEG: differentially expressed genes.

Given the distinct physiology and morphology of Mtb-ZLM vs. Mtb-ZRM (e.g., cell wall) (8), the differences in the functional macrophage responses to Mtb-ZLM vs. Mtb-ZRM (Fig. 1 and 2), and separated clusters in PCA (Fig. S3B), we hypothesized that Aux-ZLM would cause different transcriptional responses in macrophages compared with Aux-ZRM. We compared the DEGs from macrophages infected with Aux-ZLM vs. Aux-ZRM at 4 and 24 hpi to determine whether there are differences in transcriptional responses and, if so, identify genes and pathways that are differentially regulated in infected macrophages in response to these two distinct Mtb populations. At 4 hpi, there were 278 DEGs, most upregulated (244), in macrophages infected with Mtb-ZLM vs. macrophages infected with Mtb-ZRM (Fig. 4A; Table S14). Several cytokines and chemokines were upregulated, including *Il1a*, *Lif*, and *Cxcl2*. To validate these findings, enzyme-linked immunosorbent assays (ELISA) were performed to quantify protein expression of IL-1α by RAW 264.7 macrophage-like cells and BMDMs infected with Aux-ZRM and Aux-ZLM. There was an increased protein expression of IL-1α by RAW 264.7 macrophages and BMDMs infected with Aux-ZLM vs. ZRM at 4 hpi (Fig. S4). There was a smaller number of DEGs at 24 hpi, that is, there were 83 DEGs, also mostly upregulated (56), in macrophages infected with Aux-ZLM vs. Aux-ZRM (Fig. 4B; Table S15). Similar to 4 hpi, pro-inflammatory genes were upregulated (e.g., *Il1a*, *Il1b*, *Lcn2*, and *Ccl7*) at 24 hpi. Thus, Aux-ZLM induce more robust pro-inflammatory responses in RAW 264.7 macrophages compared with Aux-ZRM.

**Fig 5:**
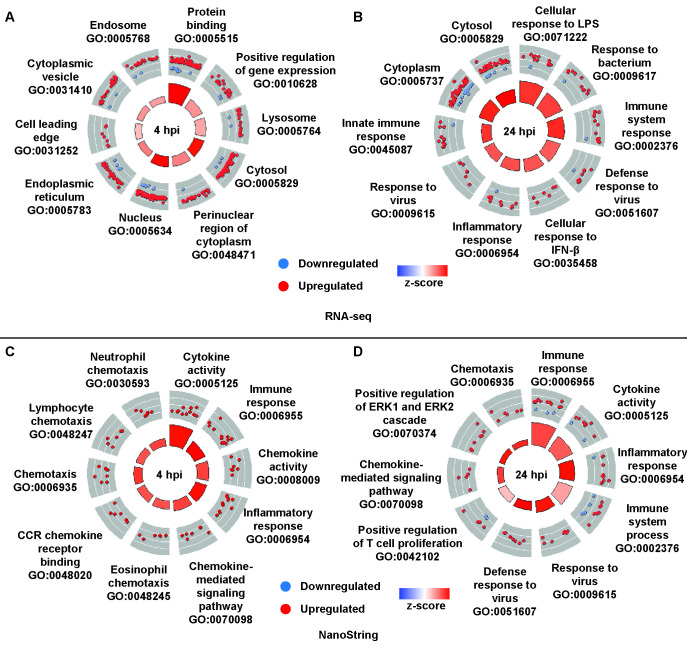
Mtb-ZLM trigger more robust inflammatory responses in RAW 264.7 macrophages compared with Mtb-ZRM. The top 10 GO terms from macrophages infected with Aux-ZLM vs. Aux-ZRM depicted with GO circle plots at (A) 4 hpi and (B) 24 hpi to show the regulation of the DEGs, as determined by RNA-seq. The top 10 GO terms from macrophages infected with Rv-ZLM vs. Rv-ZRM are shown with circle plots at (C) 4 hpi and (D) 24 hpi, as determined by NanoString. The bars in the circle plots represent the −log_10_ (FDR) and the z-scores. Taller bars have higher −log_10_ (FDR) values. The color of the bars is the z-score, which is a measure of a term’s predicted activation (red) or inhibition (blue). FDR: false discovery rate. GO: gene ontology. LPS: lipopolysaccharide. IFN: interferon. For other abbreviations See Fig. 1.

We performed IPA and KEGG pathway analyses to determine the pathways modulated in macrophages infected with Aux-ZLM vs. Aux-ZRM at 4 hpi. There was predicted activation of the “Pathogen Induced Cytokine Storm Signaling” pathway, which reflects the increased expression of pro-inflammatory cytokines in macrophages infected with Aux-ZLM vs. Aux-ZRM (Fig. 4C; Fig. S5; Table S16). KEGG pathway analysis did not show any statistically significant pathways for this comparison. GO enrichment analysis showed enrichment of DEGs and predicted activation of GO terms associated with phagocytosis (e.g., lysosome, endosome) (Fig. 5A). Note that the DEGs were commonly associated with multiple GO terms, emphasizing the complexity of the transcriptional response (Fig. S6 and S7). In summary, there was upregulation of pro-inflammatory responses in macrophages infected with Aux-ZLM vs. Aux-ZRM at 4 hpi.

Next, we applied pathway enrichment analyses to macrophages infected with Aux-ZRM and Aux-ZLM at 24 hpi. IPA showed numerous differentially modulated pathways in response to Aux-ZLM vs. Aux-ZRM (Table S17). Interestingly, the top activated pathway was the “Pyroptosis Signaling Pathway” (Fig. 4C; Fig. S8; Table S17). KEGG pathway analysis of the DEGs in macrophages infected with Aux-ZLM vs. Aux-ZRM at 24 hpi showed that there was enrichment of DEGs in two pathways: IL-17 signaling (mmu04657) and TNF signaling (mmu04668) (Fig. S9 and S10; Table S18). Tumor necrosis factor alpha (TNF-α) is implicated in Mtb pathogenesis and can be protective or detrimental to the host (24); hence, we investigated this pathway further. There were seven upregulated DEGs in this pathway, which were *Ccl2*, *Il1b*, *Lif*, *Socs3*, *Mmp9*, *Ptgs2*, and *Tnfrsf1b* (Table S18). We mapped the DEGs onto the KEGG pathway and observed that TNF-α signaling directly induces their expression, except for *Tnfrsf1b* (Fig. S10). Based on these results, we hypothesized that there could be increased production of TNF-α, despite its gene (*Tnf*) not being differentially expressed in macrophages infected with Aux-ZLM vs. macrophages infected with Aux-ZRM at 4 and 24 hpi in the RNA-seq data. We quantified secretion of TNF-α by uninfected and infected RAW 264.7 macrophages at 4 and 24 hpi using ELISA. As predicted, there was an increase in TNF-α at 4 hpi when infected with Aux-ZLM vs. Aux-ZRM (Fig. S11). The GO enrichment analysis also showed enrichment of DEGs in and predicted activation of GO terms associated with pro-inflammatory responses (e.g., cellular response to IFN-β) (Fig. 5B). Again, DEGs were associated with multiple GO terms (Fig. S12 and S13). Taken together, Aux-ZLM upregulated macrophage genes involved in pro-inflammatory pathways compared with Aux-ZRM at both early (4 hpi) and later (24 hpi) time points of the infection.

### Virulent Zn^2+^-limited Mtb also induce more robust inflammatory responses in macrophages

We sought to determine if virulent Rv-ZLM trigger increased inflammatory responses, as seen with avirulent Aux-ZLM. RAW 264.7 macrophages were infected with Rv-ZRM and Rv-ZLM, macrophage RNA was isolated at 4 and 24 hpi, and transcriptomics was done using the NanoString nCounter “mouse host response panel,” a probe-based mRNA quantification assay that covered 785 genes (25). The NanoString results are not directly comparable with the RNA-seq results due to differences in methodology between the two techniques (e.g., probe-based vs. sequencing-based, gene panel vs. all genes) (25). In agreement with the RNAseq data presented above, we observed that most genes were upregulated in macrophages infected with Rv-ZLM vs. Rv-ZRM. At 4 hpi, there were 26 DEGs, with most upregulated (21), including modest upregulation of *Tnf* (Table S19). At 24 hpi, there were 36 DEGs, with most upregulated (29) (Table S20). Although the small number of genes did not allow for IPA, we were able to employ KEGG pathway and GO enrichment analyses to determine differentially modulated pathways to determine if Aux-ZLM and Rv-ZLM induce the same phenotype, that is, increased pro-inflammatory responses. At 4 hpi, KEGG pathway and GO enrichment analyses showed enrichment of DEGs in pro-inflammatory signaling pathways (e.g., TNF-signaling pathway) (Table S21; Fig. 5C). At 24 hpi, KEGG pathway and GO enrichment analyses showed enrichment of DEGs in and predicted activation of pro-inflammatory responses (e.g., NFκB signaling pathway) (Table S22; Fig. 5D). In summary, the virulent Rv-ZLM, as seen with the attenuated strain, induce more robust pro-inflammatory responses in RAW 264.7 macrophages compared to Rv-ZRM.

## DISCUSSION

Our results suggest that Zn^2+^-limited population of *M. tuberculosis* is an important, but neglected player in TB pathogenesis. *M. tuberculosis* bacteria (Mtb) experience Zn^2+^ limitation *in vivo* in the extracellular milieu of necrotic granulomas, and Zn^2+^-limited Mtb have been detected in sputa from patients with active TB (8, 10, 11). We previously demonstrated that Mtb grown in Zn^2+^-limited medium (Mtb-ZLM) are physiologically distinct and are more virulent than Mtb grown in standard Zn^2+^-replete medium (Mtb-ZRM) in the mouse infection model (8). However, the host responses to this highly relevant population of Mtb were not addressed. In this study, we focus on how macrophages respond to Zn^2+^-limited Mtb.

**Fig 6:**
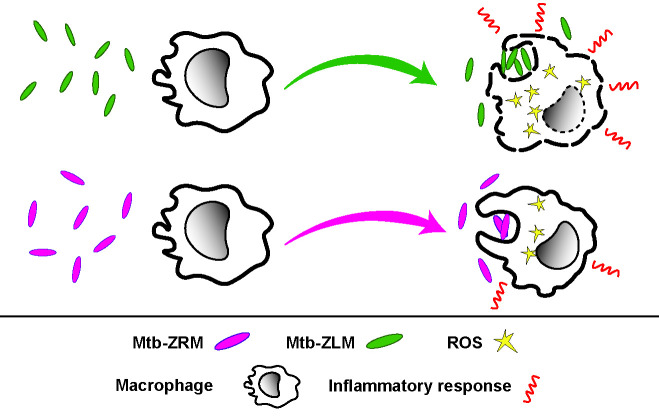
*In vitro* model of macrophage response to zinc-limited vs. zinc-replete mtb. Macrophages phagocytose more Mtb-ZLM vs. Mtb-ZRM, which triggers increased production of ROS in macrophages infected with Mtb-ZLM vs. Mtb-ZRM. Macrophages exposed to Mtb-ZLM are also more likely to die (dashed line) than those infected with Mtb-ZRM. Macrophages upregulate pro-inflammatory responses (red lines) when infected with Mtb-ZLM vs. Mtb-ZRM. Mtb-ZRM and Mtb-ZLM: Zn^2+^-replete and Zn^2+^-limited Mtb. ROS: reactive oxygen species.

Macrophages are one of the main host cells infected by Mtb and have critical roles in Mtb pathogenesis (4). Mtb produce many factors (e.g., lipids, proteins) that can be recognized by macrophages and shape macrophage responses (13). Therefore, given the remodeling of proteomes and lipidomes triggered by Zn^2+^-limitation (8), we aimed to characterize the macrophage responses to Mtb-ZLM and compare them with the responses to the standard Mtb-ZRM. Our study demonstrates more robust phagocytosis, increased ROS production, more macrophage death, and increased inflammatory responses in macrophages infected with Mtb-ZLM vs. macrophages infected with Mtb-ZRM (Fig. 6). Therefore, these findings highlight the need for researchers to consider the physiology of Zn^2+^-limited Mtb when characterizing host-pathogen interactions.

The distinct cell wall morphology and composition of Mtb-ZLM suggested that there could be modulation of phagocytosis of Mtb-ZLM vs. Mtb-ZRM (8). Here, we have shown that Mtb-ZLM are more readily phagocytosed than Mtb-ZRM, causing higher bacterial burden. Curiously, there was a more pronounced effect on the bacterial burden compared with the number of infected macrophages when phagocytosis was inhibited using CytoD. It is possible that treatment with CytoD inhibits the engulfment of aggregates of Mtb but does not affect phagocytosis of non-aggregated Mtb. This model is in agreement with our previous observations, that is, Aux-ZLM readily form aggregates, whereas Aux-ZRM do not (8). Our study is in agreement with other studies that show mycobacterial physiology can affect phagocytosis. Macrophages more readily phagocytose phosphate-limited vs. phosphate-replete *Mycobacterium bovis* BCG, a closely related species to Mtb (26). A more distant relative of Mtb, *Mycobacterium abscessus*, has rough and smooth morphotypes, with the former being phagocytosed more (usually as clumps) vs. the latter (27, 28). Thus, given that we observed differences in the uptake of Mtb-ZLM vs. Mtb-ZRM and that the physiology of other mycobacteria affects macrophage responses, one can hypothesize that the exposure to stressors prepares Mtb and other pathogenic mycobacteria for future encounters with macrophages, as we proposed previously (8). Increased uptake of Mtb-ZLM could affect several aspects of pathogenesis, as it may increase seeding of the lungs during transmission and re-infection and promote entry into the intracellular phase of the Mtb life cycle, that is, phagocytosis by macrophages and other phagocytes in necrotic granulomas.

Our previous finding that Mtb-ZLM are more resistant to oxidative stress than Mtb-ZRM suggested that Mtb-ZLM “anticipate” the impending oxidative stress upon phagocytosis (8). Our study shows that macrophages infected with Mtb-ZLM produce more ROS compared with Mtb-ZRM, which is abolished when phagocytosis is inhibited. The increases in ROS in macrophages infected with Aux-ZLM vs. Aux-ZRM at 4 hpi in the absence of CytoD are consistent with the increased phagocytosis of Aux-ZLM because there should be an increased assembly of NADPH oxidase when there is increased phagocytosis (17, 29, 30). Therefore, the increased production of ROS in macrophages infected with Aux-ZLM vs. Aux-ZRM is primarily due to phagocytosis. In addition, increased tolerance of Mtb-ZLM to ROS, as previously demonstrated (8), coupled with our findings that Mtb-ZLM-associated macrophage responses favors host cell destruction, shows that indeed the “anticipatory” adaptations in response to Zn^2+^ limitation may prime Mtb for survival in the intracellular environment and promote bacterial dissemination.

Host cell death has an integral role in Mtb pathogenesis, in which apoptotic cell death is generally beneficial to the host. In contrast, non-apoptotic cell death (i.e., necrosis) is detrimental to the host (19). In our study, we demonstrated that Mtb-ZLM induce increased death of RAW 264.7 macrophages, THP-1 macrophages, and BMDMs compared with Mtb-ZRM using both Mtb mc^2^6206 and Mtb H37Rv. However, in contrast to the ROS production, phagocytosis is not required for triggering macrophage death under conditions tested here, which is in agreement with a previous finding that extracellular Mtb can induce macrophage death via pyroptosis and that death is increased even more when macrophages interact with extracellular aggregates of Mtb (23). In addition, a high bacterial load of Mtb in macrophage can induce macrophage death mediated by permeabilization of the lysosome and release of lysosomal hydrolases (21). It is possible that the increased macrophage death caused by Mtb-ZLM was due to the prolonged contact between macrophages extracellular Mtb-ZLM, the high bacterial load of macrophages infected with Mtb-ZLM, or a combination of these two factors. The increased propensity of Zn^2+^-limited Mtb to trigger cell death may work synergistically with the neutrophilic inflammatory response to potentiate necrosis of granulomas, which often precedes lung cavitation (31), thus ultimately promoting the dissemination and transmission of Mtb.

Both Mtb-ZLM and Mtb-ZRM induce upregulation of genes involved in pro-inflammatory responses in macrophages at 4 hpi, which is consistent with numerous previous transcriptional studies with macrophages infected with Zn^2+^-replete Mtb (e.g., 32–34). Here, we also report the macrophage responses to Mtb-ZLM and Mtb-ZRM when the extracellular bacteria are not removed after 4 h of infection, that is, macrophages are exposed to extracellular Mtb for 24 h. Although at 4 hpi, there is a broad upregulation of genes involved in pro-inflammatory responses, at 24 hpi, macrophages strongly upregulate pathways involved in type I interferon signaling and the CGAS-STING signaling pathway. Indeed, it has been demonstrated that type I interferons and the CGAS-STING signaling pathway are activated when Mtb gain access to the cytosol (35–37), and when macrophages are infected at high MOIs (34). These results suggest that there could be increased DNA in the cytosol (38), and Mtb may access the cytosol when macrophages are infected at a high MOI for an extended period of time, as done in this study.

We determined that Mtb-ZLM induce more robust changes in gene expression in macrophages compared with Mtb-ZRM, particularly with the inflammatory response and at the earlier time point. The increased stimulation of macrophages by Mtb-ZLM may have been mediated by the altered cell wall and lipidome of Mtb-ZLM vs. Mtb-ZRM, which we previously reported (8). Mtb-ZLM have altered abundances of many lipids, including mycolic acids, cord factor, phosphatidyl inositol mannosides (PIMs), and phthiocerol dimycocerosate (PDIM) precursors (8). It has been shown that PIMs can inhibit TLR signaling (39, 40), whereas PDIMs can reduce the pro-inflammatory response under certain conditions (41). The observed lower abundances of PIMs and PDIMs in Mtb-ZLM vs. Mtb-ZRM support our findings which show that macrophages have increased pro-inflammatory responses to Mtb-ZLM vs. Mtb-ZRM. Therefore, Zn^2+^-limited Mtb induce more robust pro-inflammatory responses than Zn^2+^-replete Mtb, which may be driven by the altered lipidome of Zn^2+^-limited Mtb.

It is well-known that there is substantial host cell heterogeneity within granulomas during Mtb infection (42) and that there is phenotypic heterogeneity in Mtb populations *in vivo* (3). Despite the breadth of knowledge about host and bacterial heterogeneity, it is unknown if the host responds differently to populations of Mtb that are physiologically distinct, particularly the highly relevant and understudied population of Zn^2+^-limited Mtb (8). Here, for the first time, we have shown that macrophages respond differently to Zn^2+^-limited Mtb compared with the standard and well-studied Zn^2+^-replete Mtb, and we suggest that these modulated macrophage responses may have significant impacts on the host responses and outcomes of infection. Our *in vitro* macrophage infection study shows that macrophages have increased phagocytosis, production of ROS, cell death, and pro-inflammatory responses in response to Zn^2+^-limited Mtb vs. the standard Zn^2+^-replete Mtb (Fig. 6). Currently, we do not know the mechanisms by which Zn^2+^-limited Mtb cause these phenotypes. However, given that Zn^2+^-limited Mtb differentially express hundreds of genes, many of which are uncharacterized (8), it is plausible that some of these genes are virulence factors that contribute to our observed phenotypes, which could be elucidated in future studies.

Furthermore, as we previously proposed, the changes in Mtb physiology that occur in response to the Zn^2+^-limited milieu of necrotic granulomas could promote dissemination and transmission within and between hosts (8). Mtb and infected macrophages interact with other cell types that have critical roles in disease progression, including other macrophages, neutrophils, and lymphocytes (4). These different cell types could interact differently with macrophages infected with Zn^2+^-limited Mtb vs. Zn^2+^-replete Mtb or directly interact with Zn^2+^-limited Mtb and Zn^2+^-replete Mtb, thus further amplifying their specific signals. Although our experiments do not address these *in vivo* interactions, our findings emphasize that Zn^2+^-limited Mtb modulate macrophage function differently than the widely used Zn^2+^-replete Mtb, which should be considered for TB drug discovery and vaccine development.

## MATERIALS AND METHODS

### Reagents and bacterial media

Unless otherwise noted, all media and reagents were purchased from ThermoFisher Scientific or VWR. The 100% stocks of ADS were composed of 50 g/L bovine serum albumin, fraction V, pH 7 (ThermoFisher, cat # J10857-A1), 8.5 g/L dextrose, and 20 g/L sodium chloride. Middlebrook 7H9 with 10% albumin-dextrose-sodium chloride and 0.05% Tween 80 (7H9-ADS) was used for routine culturing before experiments and for generation of frozen stocks. Sauton’s medium was composed of 0.05% wt/vol KH_2_PO_4_, 0.05% wt/vol MgSO_4_7H_2_O, 0.2% wt/vol citric acid, 0.005% wt/vol ferric ammonium citrate, 6% vol/vol glycerol, 0.4% wt/vol L-asparagine, and 0.05% vol/vol Tween 80 at pH 7.4 (adjusted with freshly prepared NaOH), made with type I Milli-Q water, and filter-sterilized through 0.2 µm PES filters (VWR #10040–440). Glass containers were avoided, and media were not autoclaved in order to minimize Zn^2+^ contamination. All labware used to prepare Sauton’s medium was composed of polypropylene plastic and was rinsed at least six times with type I water before the medium was prepared. Sauton’s medium with added 6 µM ZnSO_4_ is referred to as Zn^2+^-replete medium (ZRM), and Sauton’s medium without added ZnSO_4_ is referred to as Zn^2+^-limited medium (ZLM), as previously described (43).

### Bacterial strains and plasmids

*M. tuberculosis* (Mtb) mc^2^6206 (Mtb-Aux) (44), an auxotrophic strain that cannot synthesize L-leucine and D-pantothenate, and the virulent Mtb H37Rv (Mtb-Rv) strain were used when indicated. All active manipulation of Mtb-Aux and Mtb-Rv was done in biosafety level 2 and 3 laboratories, respectively.

The fluorescent strain of Mtb-Aux expressing DsRed (pMSP12::DsRed, Addgene plasmid # 30171) and GFP (Live-Dead reporter; a gift from Dr. Christopher Sassetti) were used for the flow cytometry assays and confocal microscopy when indicated (45, 46). Antibiotics were included in 7H9-ADS when Mtb-Aux expressing DsRed (12.5 µg/mL Kanamycin) and Mtb-Aux expressing GFP (25 µg/mL Zeocin) were cultured in 7H9-ADS medium. Antibiotics were omitted from Sauton’s media. All media used to culture Mtb-Aux were supplemented with 50 µg/mL L-leucine and 50 µg/mL calcium D-pantothenate.

### Cell lines and culturing

The murine RAW 264.7 macrophage-like cell line (macrophages) was purchased from Sigma Aldrich (origin: ECACC-European Collection of Authenticated Cell Cultures). This is a transformed and immortalized cell line that was isolated from a male BALB/c mouse infected with the Abelson murine leukemia virus (47). The RAW 264.7 macrophages were routinely cultured in non-treated plastic vessels at 37°C with 5% CO_2_ in a humidified incubator and were passaged when the cell density reached 2.5 × 10^6^ to 3 × 10^6^ cells/mL (~80% confluency), as determined with the Trypan blue exclusion assay and an Invitrogen Countess (ThermoFisher Scientific #C10281). To pass the macrophages, the cells were diluted to a starting density of 2 × 10^5^ or 4 × 10^5^ cells/mL and incubated for 3 or 2 days, respectively, before being passaged again. For all assays, macrophages that were passaged at a starting density of 4 × 10^5^ cells/mL were used. The RAW 264.7 cell line was used between passages #14 and #25 (from the ECACC passage number) for all assays to limit the effects of genetic drift (48). The growth medium used was Dulbecco’s Modified Eagle Medium (DMEM) with 4.5 g/L glucose, pyruvate, and L-glutamine (Corning, cat # 10–013-CV) and 10% qualified, heat-inactivated fetal bovine serum (FBS), USA-origin (ThermoFisher Scientific #16140071). Cell viability was monitored using the Trypan Blue exclusion assay and an Invitrogen Countess instrument, and cells were only used when the viability was >90%.

The human THP-1 monocytic-like cell line (American Type Culture Collection) was a gift from Dr. Diane W. Taylor (John A. Burns School of Medicine). This immortalized cell line was isolated from a 1-year-old male with acute monocytic leukemia (49). The THP-1 cells were routinely cultured in RPMI1640 with glutagro (Corning #10–104-CV) and 10% FBS (RPMI-10% FBS) at 37°C with 5% CO_2_ in a humidified incubator and were sub-cultured when the cell density reached 0.8 × 10^6^ to 1.1 × 10^6^ cells/mL. To pass the THP-1 cells, the cells were diluted to starting densities of 1 × 10^5^ to 4 × 10^5^ cells/mL and grown for 2–4 days until the cell density reached 0.8 × 10^6^ to 1.1 × 10^6^ cells/mL. For all assays, the cells that were passed at a starting density of 400,000 cells/mL were used. The cell viability of the THP-1 cells was monitored using the Trypan Blue exclusion assay and measured using Invitrogen Countess, and the cells were only used for assays when their viability was >90%.

For both RAW 264.7 and THP-1 cell lines, the cells were only cultured in the presence of an antibiotic-antimycotic solution (ThermoFisher Scientific #15240062) when recovered from frozen stocks. They were cultured without the antibiotic-antimycotic solution for at least one week before all infection assays to prevent intracellular accumulation of the compounds.

### Preparation of RAW 264.7 macrophages and THP-1 macrophages for infection

RAW 264.7 macrophages were prepared 20–24 h before infection. Cultures of RAW 264.7 macrophages were centrifuged at 400 × *g* for 3 min and resuspended in fresh DMEM-10% FBS supplemented with 50 µg/mL L-leucine and 50 µg/mL calcium D-pantothenate. Treated tissue culture multiwell plates were used for all assays, except for flow cytometry assays in which non-treated plastic was used. We found that RAW 264.7 macrophages are extremely adherent when cultured on treated plastic and cannot be removed from treated plastic without mechanically removing them with cell scrapers, which decreases their viability. Non-treated plastic was used as an alternative for flow cytometry experiments because the cells still adhere to it but are easily removed by gentle pipet-mixing and incubation with cold PBS for 5 min without affecting macrophage viability. For colony counting assays, 3 × 10^4^ RAW 264.7 macrophages in 100 µL were added per well in treated 96-well plates 16–20 h before infection. For flow cytometry assays, 1.8 × 10^5^ RAW 264.7 macrophages in 500 µL were added per well in non-treated 24-well plates 16–20 h before infection. For the RNA-seq assay, 10^6^ RAW 264.7 macrophages in 5 mL were added per well in treated 6-well plates 16–20 h before infection. For the NanoString assay, 3.6 × 10^5^ RAW 264.7 macrophages in 1 mL were added per well in treated 12-well plates 16–20 h before infection.

THP-1 cells were grown until a density of 0.8 × 10^6^ to 1.1 × 10^6^ cells/mL was reached. The cells were centrifuged at 400 × *g* for 10 min and resuspended in RPMI-10% FBS at a density of 10^6^ cells/mL. To differentiate the THP-1 monocytic-like cells into macrophage-like cells, phorbol 12-myristate-13-acetate (PMA) (Adipogen #AG-CN2-0010-M001) was added to the cells at a final concentration of 100 nM (~62.5 ng/mL), and 100 µL of cells were added per well in treated 96-well plates 1 day before infection. On the day of infection (24 h after PMA was added and 3 h before infection), the cells were washed three times to remove PMA, and fresh 100 µL RPMI-10% FBS with 50 µg/mL L-leucine and 50 µg/mL calcium D-pantothenate without PMA was added per well.

For all macrophages, flat bottom cell culture grade multiwell plates were used, and the cell titers were determined using the Trypan Blue exclusion assay and measured using Invitrogen Countess.

### Preparation of murine bone marrow-derived macrophages for infection

Murine bone marrow-derived macrophages (BMDMs) were prepared from the tibia, femur, and pelvic bones of male and female C57BL/6 mice that were 12–16 weeks of age. Bones were collected from mice that were sacrificed by CO_2_ asphyxiation in accordance with the Institutional Animal Care and Use Committee (University of Hawaiʻi at Mānoa) exempt letter TEX-15–008. Bones were flushed with buffer (RPMI-10% FBS), and the bone marrow was centrifuged, resuspended in RPMI-10% FBS with 1% antibiotic-antimycotic, and 12% L929 conditioned medium containing M-CSF (made in-house). The cells were incubated and differentiated on bacteriological Petri dishes (pyrogen-free) for 5 days at 37°C with 5% CO_2_ in a humidified incubator. After 5 days of differentiation (and 1 day before infection), the cells were washed to remove non-differentiated and contaminating cells, and the BMDMs were lifted off using CellStripper, centrifuged, and resuspended in RPMI-10% FBS without antibiotic-antimycotic and L929 conditioned medium. BMDMs were diluted to 10^6^ cells/mL and added to 96-well (100 µL) or 24-well plates (500 µL). Cell viability was measured using Trypan Blue exclusion assays and an Invitrogen Countess instrument, and BMDMs were only used if viability was >90%.

### Preparation of Zn^2+^-replete and Zn^2+^-limited Mtb cultures for infection

Mtb-Aux and Mtb-Rv grown in ZLM and ZRM in biological triplicates were prepared as described previously (8). Due to additional restrictions in the BSL3 laboratory, all liquid cultures of Mtb-Rv were kept in Ziploc bags. All liquid cultures of Mtb-Aux were not bagged (i.e., fully aerated). Mtb-Aux grown in ZLM and ZRM are referred to as Aux-ZLM and Aux-ZRM, and Mtb-Rv grown in ZLM and ZRM are referred to as Rv-ZLM and Rv-ZRM. Briefly, 1 mL frozen stocks of Mtb-Aux or Mtb-Rv were inoculated into 5 mL of 7H9-ADS in 15 mL snap-cap tubes (Mtb-Aux) or 50 mL bioreactor tubes (Mtb-Rv) and incubated for at least 1 week at 37°C without shaking until the optical density at 600 nm (OD_600_) reached ~1. They were subcultured by inoculating 1 mL of culture into 50 mL of 7 H9-ADS in 250 mL vented, polycarbonate flasks, incubated at 37°C with 120 rpm shaking for 5 days until OD_600_ 0.5–0.8. Cultures were centrifuged and resuspended in ZLM two times and diluted to OD_600_ 0.08 after the last wash step. To prepare cultures in ZRM, ZnSO_4_ was added to a final concentration of 6 µM. Cultures in Sauton’s medium were grown for 8 days to achieve Zn^2+^ limitation, and Zn^2+^ limitation was determined by reading the fluorescence of coenzyme F420 at excitation and emission spectra 420 nm/475 nm, as reported previously (8, 43). Antibiotics were excluded from Sauton’s medium in case they affected bacterial growth or physiology in ZLM vs. ZRM cultures. After 8 days of growth in Sauton’s medium, the cultures were centrifuged at 800 × *g* for 10 min to pellet large clumps. Supernatants were then passed through 10 µm strainers (pluriSelect #43–50010-03) to generate clump-free cell suspensions, and OD_600_ was measured. Clump-free cell suspensions were centrifuged at 3,000 × *g* for 10 min. To normalize the colony forming units per mL (CFU/mL) of Aux-ZLM and Aux-ZRM, Aux-ZLM were resuspended at OD_600_ of 0.5, and Aux-ZRM were resuspended in serum-free DMEM or RPMI1640 at OD_600_ of 0.35. For Mtb-Rv, both Rv-ZLM and Rv-ZRM were resuspended in serum-free DMEM or RPMI1640 at OD_600_ of 0.5 to normalize CFU/mL to approximately 10^8^ CFU/ml (Fig. S14). The CFU/mL was determined by preparing dilutions of ZLM and ZRM in filter-sterilized spent Mtb media and plating onto 7H11-ADS agar plates. Clump-free cells were found to grow very poorly when plated without spent media. The plates were incubated at 37°C for 3 weeks, and the CFUs were counted.

RAW 264.7 macrophages were infected with the OD-adjusted Mtb cultures at a multiplicity of infection of 20 bacterial cells per host cell (MOI 20) by adding 12 µL of OD-adjusted Mtb-ZRM and Mtb-ZLM to 6 × 10^4^ cells in 100 µL per well in 96-well plates, 72 µL of OD-adjusted Mtb to 3.6 × 10^5^ macrophages in 500 µL per well in 24-well plates, 144 µL of OD-adjusted Mtb to 7.2 × 10^5^ macrophages in 1 mL per well in 12-well plates, and 400 µL of OD-adjusted Mtb to 2 × 10^6^ macrophages in 5 mL per well in 6-well plates. The approximate macrophages per well were determined using an estimated 16 h doubling time of the RAW 264.7 cell line. BMDMs and THP-1 macrophages were infected at MOI 10 by adding 10 µL of OD-adjusted Mtb to 1 × 10^5^ and 5 × 10^5^ macrophages per well in 96-well and 12-well plates, respectively. The lower MOI was used for BMDMs and THP-1 cells because these cells were dying much faster than RAW 264.7 cells at MOI 20.

### Colony forming unit (CFU) uptake assays of Mtb

For uptake assays in RAW 264.7 macrophages, the macrophages were infected with Mtb-Aux and Mtb-Rv grown in ZLM and ZRM at MOI 20 for 24 h. Intracellular Mtb were determined at 4 and 24 hpi to measure the uptake of Mtb. The macrophages were washed three times with Hank’s Balanced Salt Solution to remove the extracellular bacteria (removal confirmed via microscopy) before lysis. Macrophages were lysed by incubation with 0.05% sodium dodecyl sulfate (wt/vol) for 5–10 min, confirmed to be lysed by microscopy, and macrophage lysates were collected. The wells that contained the macrophage lysates were washed three times using 7H9-ADC to gather all remaining Mtb. The lysate and washes were pooled and serially diluted. The final serial dilutions were prepared in spent Mtb media, plated onto 7H11-ADS agar plates, and incubated at 37°C. The plates were incubated at 37°C for 3 weeks, and the CFUs were counted.

### Flow cytometry to determine phagocytosis of Mtb by RAW 264.7 macrophages

Mtb expressing DsRed or GFP was used to infect RAW 264.7 macrophages. RAW 264.7 macrophages were infected at MOI 20 with Mtb-ZRM and Mtb-ZLM, and macrophages were harvested at 4 and 24 hpi. The macrophages were centrifuged at 400 × *g* for 8 min at 4°C and resuspended in 500 µL of cold RPMI with 2% FBS and 25 mM HEPES. Samples were kept on ice before flow cytometry. All assays were done within 1 h of sample preparation. The cells were analyzed on an Attune NxT flow cytometer (Molecular and Cellular Immunology Core at the John A. Burns School of Medicine). Two microliters of DRAQ7 DROP & GO Dye (ThermoFisher, cat #D15107) were immediately added to each sample before analysis on an Attune NxT flow cytometer. Macrophage singlets were gated (Fig. S15A), and the YL2 channel of the yellow laser was used to measure the abundance of DsRed-negative (−) and DsRed-positive (+) macrophage populations, and the median fluorescence intensity (MFI) of DsRed (Fig. S15B). The data were exported as .fcs files and analyzed using FlowJo v10.8.1 software (BD Biosciences). The median fluorescence intensities were measured in DsRed-(+) macrophages to determine bacterial burden.

### Measurement of fluorescence intensity of DsRed-expressing and GFP-expressing mtb

DsRed-expressing and GFP-expressing Mtb-Aux were grown in ZRM and ZLM for 8 days, clump-free cell suspensions were prepared, and OD_600_ was adjusted to normalize CFUs/mL, as described above. Fluorescence intensities of DsRed and GFP from OD_600_-adjusted Aux-ZRM and Aux-ZLM and blanks (Sauton’s medium) were measured using excitation-emission spectra of 550 nm/590 nm and 480 nm/520 nm using a Tecan Infinite M200 pro plate reader.

### Confocal microscopy used to determine phagocytosis of Mtb by RAW 264.7 macrophages

Confocal microscopy assays were done using Mtb expressing GFP via the Live-Dead reporter and Millicell EZ slides (MilliporeSigma #PEZGS0816). RAW 264.7 macrophages were seeded at a density of 4 × 10^5^ live cells per well 24 h prior to infection, and 48 µL of OD600-normalized Mtb was added per well to achieve an MOI of 20. Anhydrotetracycline was added to a final concentration of 200 ng/mL at the time of infection to attempt to induce RFP expression by live Mtb-Aux. It was found that intracellular Aux-ZRM and Aux-ZLM were unable to express RFP when anhydrotetracycline was added to macrophage infection assays (data not shown). Sterile ZRM and ZLM were added at equivalent volumes to uninfected RAW 264.7 macrophages as controls. At 4 and 24 hpi, extracellular bacteria were removed by repeated washing, the samples were fixed with 4% formaldehyde (methanol-free) for 10 min and washed three times with PBS to remove residual formaldehyde. Macrophage F-actin was stained with Alexa Fluor-647 conjugated to phalloidin following the manufacturer’s protocol for formaldehyde-fixed cells (Invitrogen #A22287). Briefly, macrophages were permeabilized using 0.1% Triton X-100 for 10 min, washed three times with PBS, stained with 160 nM phalloidin, and washed again. Macrophage nuclei were stained using 20 nM Hoechst 33342 for 10 min (ThermoFisher #62249). Samples were dried, mounted using ProLong Diamond AntiFade Moutant (Invitrogen #P36965), sealed, and kept at 4°C until analysis. Confocal Z-stacks were acquired using a Leica SP8 X Confocal Laser Scanning Microscope (Biological Electron Microscope Facility at the University of Hawaii). Lasers were used to detect the different fluorophores (Hoechst, GFP, RFP, Alexa Fluor 647). The data were exported as .lif files and analyzed using Fiji to export single stacks and videos (50). The contrast and brightness of the different channels were adjusted to improve the entire images for viewing purposes, but not for any quantitative analyses.

### Production of reactive oxygen species in RAW 264.7 macrophages

RAW 264.7 macrophages were seeded into 12-well plates composed of non-treated plastic and incubated for 24 h before infection. Non-fluorescent and GFP-expressing Aux-ZRM and Aux-ZLM were added to BMDMs and RAW 264.7 macrophages at MOIs 10 and 20, respectively, in technical duplicates and infected for 24 h. Uninfected macrophages were given sterile medium and were used as controls. Following 24 h, uninfected controls were treated with 1 mM N-acetylcysteine (NAC) followed by 200 µM tert-butyl hydroperoxide (TBHP) or treated with 200 µM TBHP alone. Controls were treated with NAC for 1 h at 37°C with 5% CO_2_ and treated with TBHP for 30 min at 37°C with 5% CO_2_. After treatment with TBHP, all controls and infected macrophages were stained with 2000 nM CellROX Orange for 1 h at 37°C with 5% CO_2_. The samples were then put onto ice, gently pipet-mixed to dissociate the macrophages from the plastic and transferred into tubes. Cold CellStripper (Corning #25–056-CI) was used to dissociate any macrophages that remained adherent to the plastic. RAW264.7 macrophage samples were centrifuged at 400 × *g* for 8 min at 4°C, washed once with cold CellStripper, and resuspended in cold RPMI with 2% FBS and 25 mM HEPES. BMDM samples were not washed. Two microliters of DRAQ7 were added to each sample immediately before analysis on the flow cytometer. The macrophage singlets and DRAQ-negative macrophages were gated on (Fig. S15A and C). The CellROX Orange fluorescence from the population of DRAQ-negative macrophages was determined using channel YL1 of the yellow laser (Fig. S15C). The data were exported as .fcs files and re-analyzed using FlowJo v10.8.1 software to determine statistics (e.g., percentage of cells, median fluorescence intensities, etc.).

An additional set of controls and infected macrophages were included in all assays but were not stained with CellROX Orange and instead treated with the equivalent concentration of DMSO that the CellROX-stained cells were exposed to (0.04%). These samples were included to determine autofluorescence, which was then subtracted from stained samples' median fluorescence intensities (MFIs). The unstained samples were done in technical duplicates.

### Measurement of macrophage death using flow cytometry and lactate dehydrogenase (LDH) release assays

Cell death was measured using flow cytometry and LDH release assays. For flow cytometry assays, cell death was measured using the dead cell stain, DRAQ7. Macrophage singlets were gated on and the percentages of DRAQ7-positive cells at 4 and 24 hpi were measured (Fig. S15A and D). For LDH release assays, supernatants from lysed macrophages, uninfected macrophages, and macrophages infected at MOI 20 for RAW 264.7 and at MOI 10 for THP-1 were collected at 24 hpi. For infections with Mtb-Aux, the LDH release was measured using the CyQUANT LDH Cytotoxicity Assay, per the manufacturer’s protocol (ThermoFisher Scientific #C20300). Briefly, 10× lysis buffer was added to uninfected macrophages at a final concentration of 1× to serve as the maximum LDH release control and incubated until the macrophages were lysed, which was confirmed by microscopy. Equal volumes of supernatant and Reaction Mixture were mixed, incubated for 30 min at room temperature, and one original volume of Stop Solution was added. The absorbances at 490 nm and 680 nm were measured using a Tecan Infinite M200 pro plate reader (Tecan). The A680 values were subtracted from the A490 values.

For infections with Mtb-Rv, the same controls were used, as described above, and supernatants from infected macrophages were collected at 24 hpi. The CytoTox-ONE Homogeneous Membrane Integrity Assay (Promega #G7890) was used to measure the LDH release, following the manufacturer’s protocol with some modifications. Two microliters of lysis buffer were added to uninfected macrophages and incubated for 5–10 min to lyse the cells and generate the maximum LDH release control. Fifty microliters of supernatant and 50 µL of substrate were mixed per well in flat-bottom 96-well plates and incubated for 10 min at room temperature, following which 25 µL of stop solution was added per well. The fluorescence was measured using an excitation filter of 550 nm and an emission filter of 600 nm on a Tecan GENios plate reader (Tecan).

For all LDH release assays, a cell culture medium was used as a blank to determine the background absorbance or fluorescence. The LDH activity per condition was made relative to the LDH activity of the lysed macrophage control and multiplied by 100 to determine the percentage of LDH release.

### Phagocytosis inhibition in RAW 264.7 macrophages

RAW 264.7 macrophages were seeded into 12-well plates composed of non-treated plastic and incubated for 24 h before infection. Mtb expressing GFP via the Live-Dead reporter was grown in ZRM and ZLM and used to infect macrophages at MOI 20 in technical duplicates. Cytochalasin D (CytoD; Invitrogen #PHZ1063) was added to a final concentration of 3 µM to macrophages approximately 30 min before infection with Mtb. Concentrations of CytoD above 3 µM were toxic to the macrophages and induced high cell death (data not shown). CytoD stocks were prepared at 10 mM in DMSO, aliquoted and stored at −20°C. DMSO (0.03%) was added to macrophages to serve as untreated controls. At the designated time points, controls (unstained, untreated, TBHP, NAC + TBHP), staining, and analyzed on an Attune NxT, as described above. Compensation was done for the BL1 and YL1 channels using GFP(−) CellROX Orange(−) macrophages, GFP(+) CellROX Orange(−) macrophages, and GFP(−) CellROX Orange(+) macrophages. The macrophage singlets and DRAQ-negative macrophages were gated on (Fig. S15A). The fluorescence of GFP was measured using the BL1 channel of the blue laser (Fig. S15E), and CellROX Orange fluorescence from the population of DRAQ7(−) macrophages was determined using YL1 channel of the yellow laser (Fig. S15F).

The data were exported as .fcs files, and the data were re-analyzed using FlowJo v10.8.1 software (BD) to determine statistics (e.g., percentage of cells, median fluorescence intensities, etc). The uptake of Mtb was determined by measuring the percentage of GFP+ (infected) macrophages and the MFI of GFP in infected macrophages. The CellROX Orange MFIs from the population of DRAQ7(−) GFP(+) macrophages were measured to determine production of ROS in infected macrophages.

An additional set of controls and infected macrophages were included in all assays but were not stained with CellROX Orange and instead treated with the equivalent concentration of DMSO that the CellROX-stained cells were exposed to (0.04%). These samples were included to determine autofluorescence, which was then subtracted from the MFIs of stained samples. The unstained samples were done in technical duplicates.

### RNA isolation for RNA-seq

Aux-ZLM and Aux-ZRM were grown in biological triplicates, and clump-free cell suspensions were made in ZLM and ZRM, respectively. RAW 264.7 macrophages were infected at MOI 20 in technical duplicate with each biological replicate of Aux-ZLM and Aux-ZRM. ZLM and ZRM were added to uninfected RAW 264.7 macrophages in triplicate to serve as controls for comparisons for Aux-ZLM and Aux-ZRM, respectively. One milliliter of TRIzol reagent (ThermoFisher Scientific #15596026) was added per well of macrophages at 4 and 24 hpi, and the extractions were collected. The extractions were centrifuged at 3,000 × *g* for 10 min at 4°C to pellet cell debris, and the supernatants containing RNA were stored at −80°C until RNA isolation. Total RNA was isolated using the PureLink RNA Mini Kit (ThermoFisher Scientific #12183018A) following the manufacturer’s protocol. The RNA was sent to GENEWIZ, where library preparation and sequencing were done on an Illumina HiSeq 4000 2 × 150 bp platform, and data were obtained in .FASTQ format.

### Differential gene expression analysis

#### Transcript quantification

The RNA-seq paired-end reads in FASTQ format were explored using FastQC and cleaned using Trimmomatic (51, 52). The cleaning procedure included trimming low-quality reads from both 3’ and 5’ ends until a base pair of Phred quality score of 30 (99.9% accurate) or greater was found, and filtering out reads having a mean quality score of less than 30 and lengths below 20 nucleotides. The house mouse (*Mus musculus*) reference genome (GRCm38) was indexed and aligned against HiSAT2 (53). The resulting SAM (Sequence Alignment and Mapped) files were sorted and converted to BAM files using SAMtools, and then used to generate count reads using HTSeq (54, 55).

#### Identify differentially expressed transcripts between groups

Differential gene expression analysis was performed by the DESeq2 Bioconductor package (56). The genes with Benjamini and Hochberg multiple testing q-value <0.05 and a cut-off with >0.585 and <−0.585 log_2_ fold change (i.e., >1.5 and <−1.5 fold change) were called differentially expressed.

### RNA isolation for NanoString and analysis

Rv-ZLM and Rv-ZRM were grown in biological triplicate, clump-free cell suspensions were prepared in serum-free DMEM, and RAW 264.7 macrophages were infected at MOI 20. Serum-free DMEM was added to uninfected RAW 264.7 macrophages for the uninfected control. TRIzol extractions were prepared, and RNA was isolated, as described above. The purified RNA samples were shipped to NanoString Technologies in Seattle, USA. The samples were run on the “mouse host response” nCounter panel, and the output .RCC files were analyzed using the nSolver 4.0 software (NanoString Technologies). The raw data per time point were analyzed separately (i.e., data from Rv-ZLM and Rv-ZRM at 4 hpi were analyzed separately from their 24 hpi counterparts). Genes with *P*-values < 0.05 and fold changes > 1.5 and <−1.5 were considered to be differentially expressed.

### Generation of the principal component analysis (PCA) plot

The PCA plots of the RNA-seq data were generated using DEBrowser v1.24.1 (57). The normalized count data were inputted into DEBrowser, and the default settings were used. The normalization method was set to “none,” the adjusted p-value was set to <0.05, and the fold change was set to >1.5 and <−1.5.

### Protein quantification of IL-1α and TNF-α

Cell culture supernatants and cell lysates were collected from RAW 264.7 macrophages and BMDMs (lysates only) that were uninfected or infected with Aux-ZRM and Aux-ZLM for 4 hpi. For the cell culture supernatants, the cell culture medium was collected, centrifuged at 20,000 × *g* for 10 min, the supernatants were passed through 0.22 µm cellulose acetate spin filters (Corning #8160), and frozen at −80°C until analysis. For the cell lysates, the macrophages were washed three times with PBS to remove contaminating cytokines from the cell culture supernatant and lysed using RIPA lysis buffer (ThermoFisher Scientific #89900). The cell lysates were centrifuged at 20,000 × *g* for 10 min, the supernatants were passed through 0.22 µm cellulose acetate spin filters, and frozen at −80°C until analysis. The protein concentrations of TNF-α in the cell culture supernatants were quantified using the ELISA MAX Deluxe Set Mouse TNF-α Kit (BioLegend #430904), as described in the manufacturer’s protocol. The protein concentration of IL-1α was quantified in macrophage lysates using the IL-1α ELISA MAX Standard ELISA Kit (BioLegend #433104), as described in the manufacturer’s protocol.

### Pathway enrichment analyses

Ingenuity Pathway Analysis (IPA; QIAGEN Inc., https://digitalinsights.qiagen.com/IPA) (58) was done using the differentially expressed genes (DEGs) from the comparisons to determine pathways that were predicted to be modulated. The pathways with *P*-value < 0.05 and z-score >2 or <-2 were considered significant. KEGG pathway analyses (59–61) and GO analyses (62, 63) were done using the RNA-seq data and NanoString data using DAVID 2021 (https://david.ncifcrf.gov/) (64, 65). The DEGs were inputted into DAVID, and the data files containing KEGG pathways, and the GO terms associated with biological processes, cellular components, and molecular function were retrieved and saved as .csv files. KEGG pathways and GO terms with false discovery rates below 0.05 were deemed statistically significant. The GO analyses’ output files were formatted for analysis using GOplot to construct the circle, chord, and heatmap plots (66). DEGs were mapped onto the KEGG pathways using the KEGG Mapper tool (https://www.genome.jp/kegg/mapper/) (67, 68).

### Statistical analysis

Unpaired *t*-tests with Welch’s correction were used for most comparisons, and the Holm-Šídák method was used for multiple comparisons when applicable. A two-way ANOVA with Šídák’s multiple comparison test was done in Fig. 3D. All statistical tests were done using Prism v10.0.3 (GraphPad). All biological replicates were done in technical duplicates, unless otherwise indicated. Differences were determined to be statistically significant if the p-value was below 0.05.

## Data Availability

The RNA-seq data were deposited to the Gene Expression Omnibus (GEO) database with accession number GSE255565 (69–71). The NanoString data were deposited to the GEO database with accession number GSE256252 (69–71).

## SUPPLEMENTAL MATERIAL

Supplemental figures iai.00578-24-s0001.pdf Fig. S1 to S15.

Supplemental tables iai.00578-24-s0002.xlsx Tables S1 to S22.

Video S1 iai.00578-24-s0003.avi Confocal microscopy of RAW 264.7 macrophages infected with Aux-ZRM at 24 hpi

Video S2 iai.00578-24-s0004.avi Confocal microscopy of RAW 264.7 macrophages infected with Aux-ZLM at 24 hpi

ASM does not own the copyrights to Supplemental Material that may be linked to, or accessed through, an article. The authors have granted ASM a non-exclusive, world-wide license to publish the Supplemental Material files. Please contact the corresponding author directly for reuse.
