# Supplementary material for: Zinc-limited Mycobacterium tuberculosis stimulate distinct responses in macrophages compared with standard zinc-replete bacteria
Source: Infect Immun. 2025 Feb 4;93(3):e00578-24. doi: 10.1128/iai.00578-24 (PMC11895486; doi:10.1128/iai.00578-24)
Supplement: Supplemental figures — Fig. S1 to S15. [file iai.00578-24-s0001.pdf]

SUPPLEMENTARY FIGURES

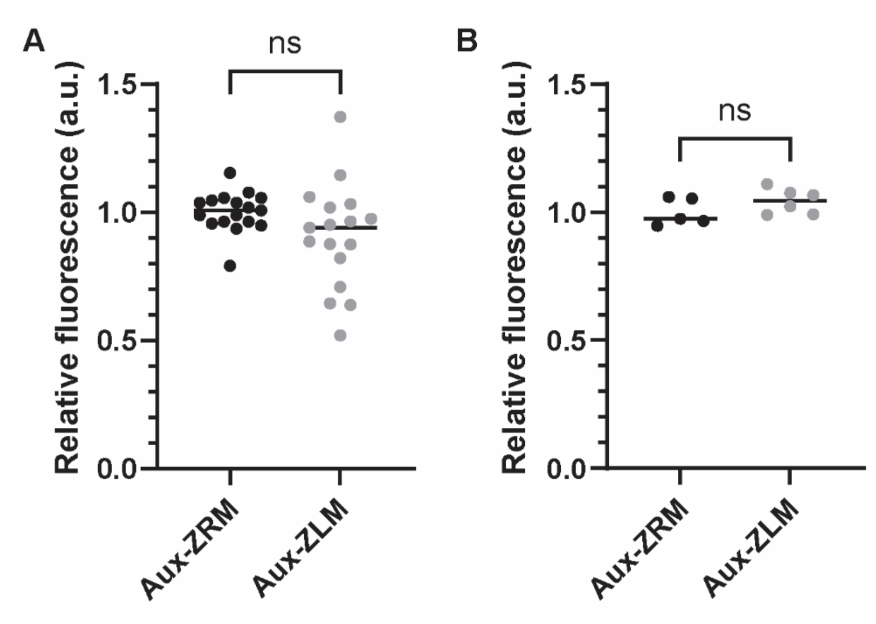

Fig S1: Relative fluorescence of Aux-ZRM and Aux-ZLM strains. A) DsRed-expressing strain and B) GFP-expressing strain. Fluorescence is relative to Aux-ZRM for each strain. All results from 6 and 2 independent assays for DsRed and GFP, respectively, are shown. ns: not significant.

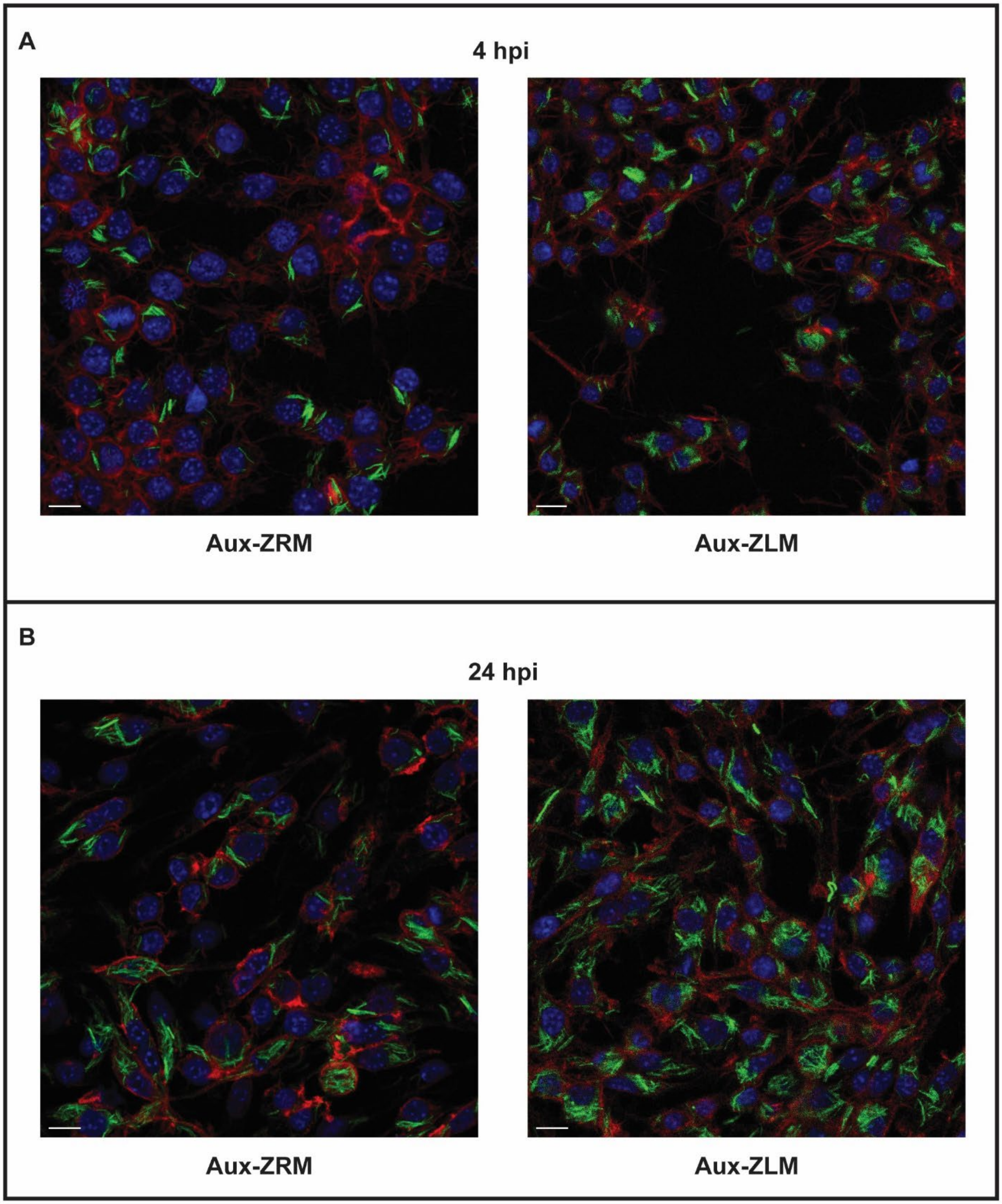

Fig S2: Representative confocal microscopy images of infected RAW 264.7 macrophages.

Macrophages were infected with fluorescent Aux-ZRM or Aux-ZLM, as indicated, and

visualized at A) 4 hpi and B) 24 hpi. Mtb-Aux expressed GFP via the LiveDead reporter plasmid were mostly localized closed to nuclei (blue) and associated with actin (red). Single stacks were exported, and contrast and brightness were adjusted for visual purposes only. Scale bar is 10 μm.

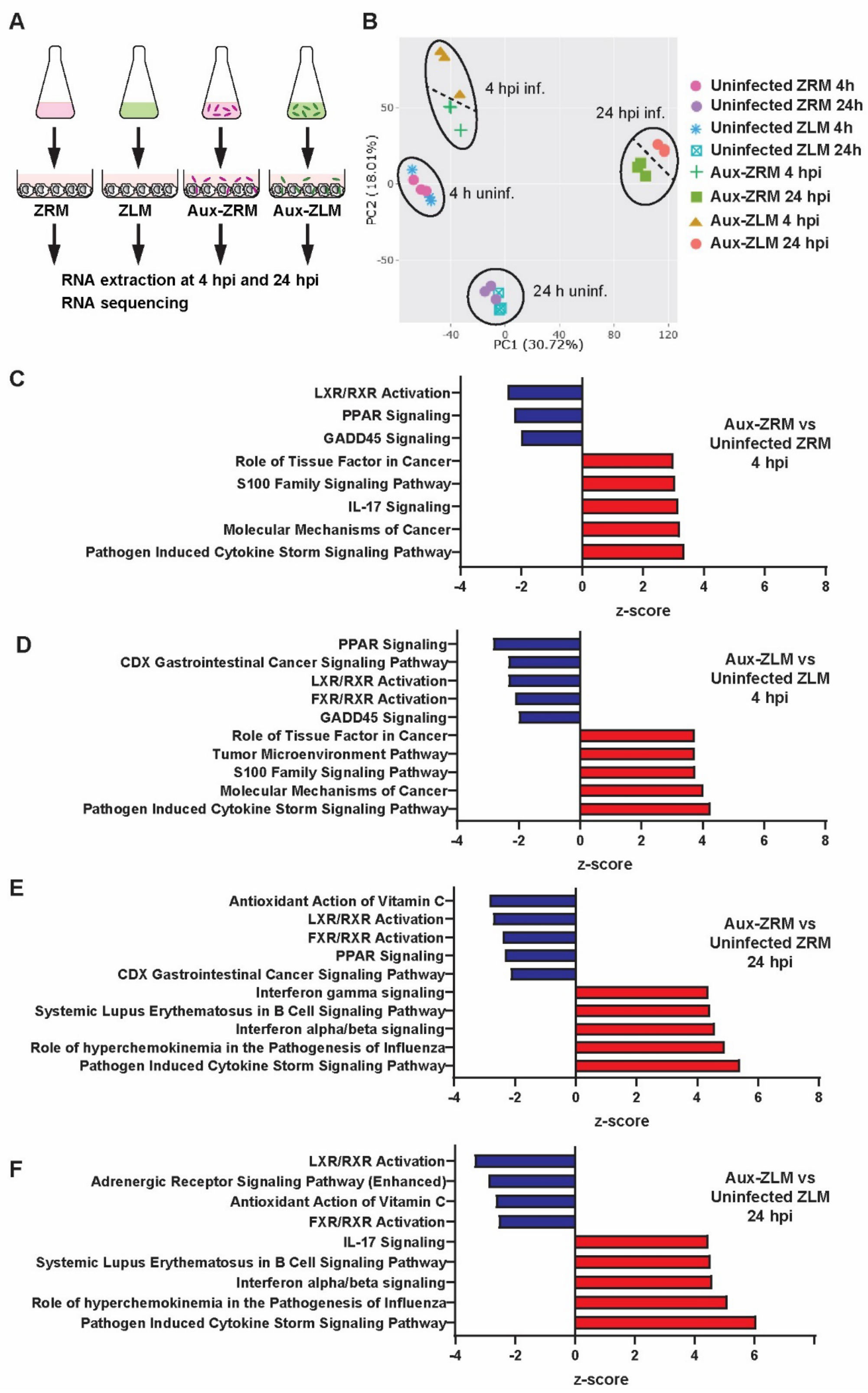

A
ZRM
ZLM
Aux-ZRM
Aux-ZLM
RNA extraction at 4 hpi and 24 hpi
RNA sequencing
B
4 hpi inf.
24 hpi inf.
4 h uninf.
24 h uninf.
PC1 (30.72%)
PC2 (18.01%)
Uninfected ZRM 4h
Uninfected ZRM 24h
Uninfected ZLM 4h
Uninfected ZLM 24h
Aux-ZRM 4 hpi
Aux-ZRM 24 hpi
Aux-ZLM 4 hpi
Aux-ZLM 24 hpi
C
LXR/RXR Activation
PPAR Signaling
GADD45 Signaling
Role of Tissue Factor in Cancer
S100 Family Signaling Pathway
IL-17 Signaling
Molecular Mechanisms of Cancer
Pathogen Induced Cytokine Storm Signaling Pathway
Aux-ZRM vs Uninfected ZRM 4 hpi
z-score
D
PPAR Signaling
CDX Gastrointestinal Cancer Signaling Pathway
LXR/RXR Activation
FXR/RXR Activation
GADD45 Signaling
Role of Tissue Factor in Cancer
Tumor Microenvironment Pathway
S100 Family Signaling Pathway
Molecular Mechanisms of Cancer
Pathogen Induced Cytokine Storm Signaling Pathway
Aux-ZLM vs Uninfected ZLM 4 hpi
z-score
E
Antioxidant Action of Vitamin C
LXR/RXR Activation
FXR/RXR Activation
PPAR Signaling
CDX Gastrointestinal Cancer Signaling Pathway
Interferon gamma signaling
Systemic Lupus Erythematosus in B Cell Signaling Pathway
Interferon alpha/beta signaling
Role of hyperchemokinemia in the Pathogenesis of Influenza
Pathogen Induced Cytokine Storm Signaling Pathway
Aux-ZRM vs Uninfected ZRM 24 hpi
z-score
F
LXR/RXR Activation
Adrenergic Receptor Signaling Pathway (Enhanced)
Antioxidant Action of Vitamin C
FXR/RXR Activation
IL-17 Signaling
Systemic Lupus Erythematosus in B Cell Signaling Pathway
Interferon alpha/beta signaling
Role of hyperchemokinemia in the Pathogenesis of Influenza
Pathogen Induced Cytokine Storm Signaling Pathway
Aux-ZLM vs Uninfected ZLM 24 hpi
z-score

Fig S3: Aux-ZRM and Aux-ZLM trigger robust changes in gene expression in RAW 264.7 macrophages compared to uninfected macrophages at 4 hpi and 24 hpi. A) Schematic of RNAseq experimental design. B) Principal Component Analysis (PCA) plot of RNA-seq samples. The top 5 activated and inhibited IPA canonical pathways in C) macrophages infected with Aux-ZRM vs. uninfected macrophages with ZRM at 4 hpi, D) macrophages infected with Aux-ZLM vs. uninfected macrophages with ZLM at 4 hpi, E) macrophages infected with Aux-ZRM vs. uninfected macrophages with ZRM at 24 hpi, and F) macrophages infected with Aux-ZLM vs. uninfected macrophages with ZLM at 24 hpi. The dashed lines drawn between Aux-ZRM and Aux-ZLM at 4 hpi and 24 hpi in the PCA plot highlight the differences between the groups. 4 hpi and 24 hpi inf.: infected macrophages at 4 hpi and 24 hpi. 4 h and 24 h uninf.: uninfected macrophages at 4 h and 24 h. PPAR: Peroxisome proliferator-activated receptor. LXR: Liver X Receptor. FXR: Farnesoid X Receptor. RXR: Retinoid X Receptor. GADD45: Growth Arrest and DNA Damage.

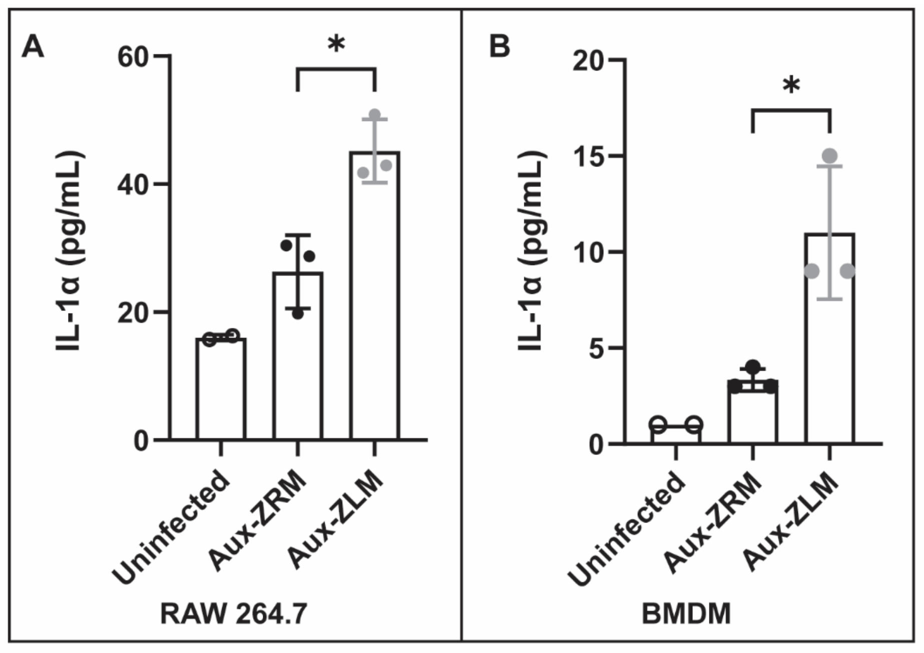

Fig S4: Quantification of IL-1α in cell lysates from A) RAW 264.7 cells and B) BMDMs that were either uninfected or infected with Aux-ZRM or Aux-ZLM at 4 hpi, as indicated. Note that the later timepoint at 24 hpi could not be compared due to the difference in cell death. There were two and three biological replicates for uninfected and infected conditions, respectively, and each biological replicate was measured in technical duplicate. Standard deviations are shown. Representative results of at least two independent experiments are shown. BMDMs: bone marrow-derived macrophages. *: p-value <0.05.

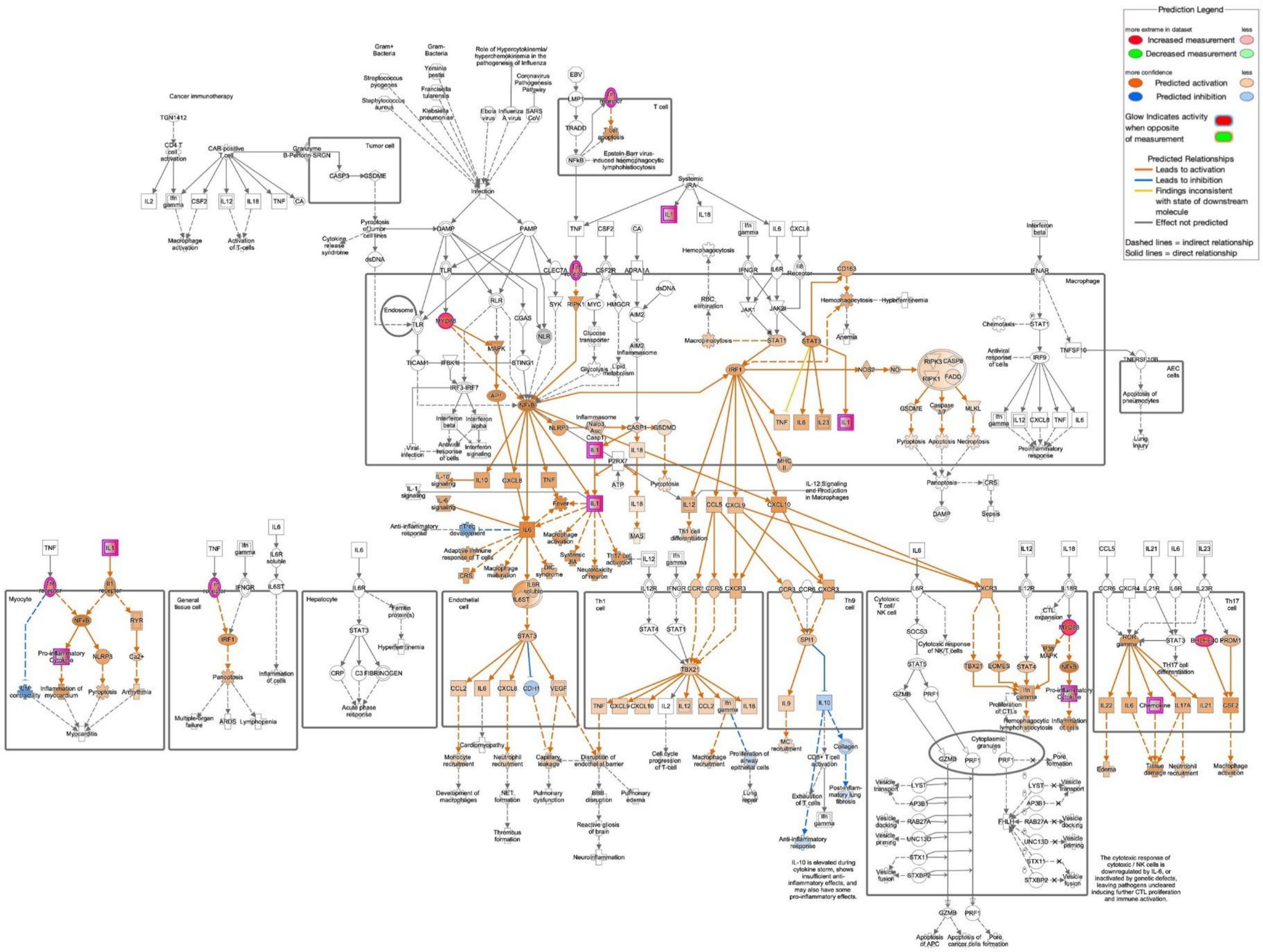
Prediction Legend
Increased measurement
Decreased measurement
Predicted activation
Predicted inhibition
Glow Indicates activity when opposite of measurement
Predicted Relationships
Leads to activation
Leads to inhibition
Findings inconsistent with state of downstream molecule
Effect not predicted
Dashed lines = indirect relationship
Solid lines = direct relationship
Cancer immunotherapy
Macrophage
T cell
Tumor cell
Myocyte
General tissue cell
Hepatocyte
Endothelial cell
Th1 cell
Th9 cell
Cytotoxic T cell/ NK cell
Th17 cell
AEC cells
IL-10 is elevated during cytokine storm, shows insufficient anti-inflammatory effects, and may also have some pro-inflammatory effects.
The cytotoxic response of cytotoxic / NK cells is downregulated by IL-6, or inactivated by genetic defects, leaving pathogens uncleared inducing further CTL proliferation and immune activation.

Fig S5: Predicted activation of the “Pathogen Induced Cytokine Storm Signaling” IPA pathway. The pathway was constructed with IPA using gene expression in RAW 264.7 macrophages infected with Aux-ZLM vs. Aux-ZRM at 4 hpi.

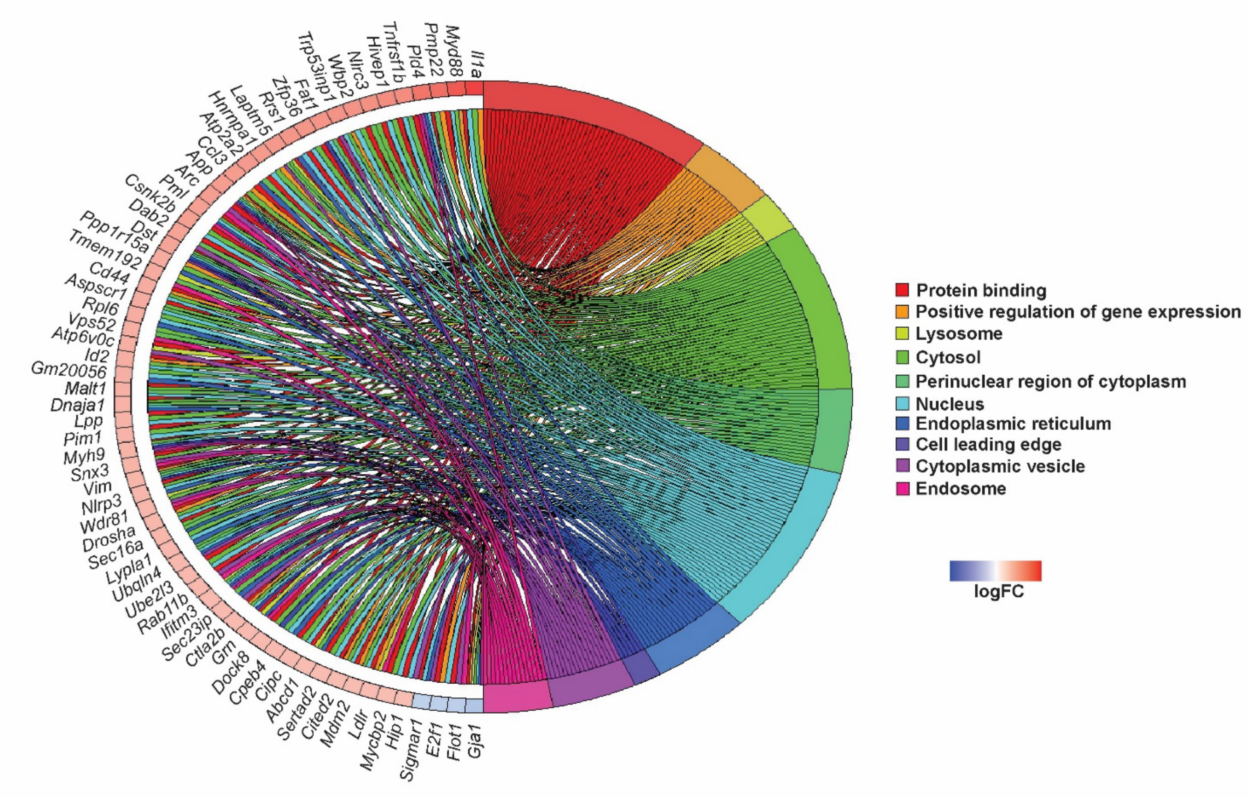

Fig S6: Chord plot of the top 10 GO terms from macrophages infected with Aux-ZLM vs. Aux-ZRM at 4 hpi. The plot was constructed using gene expression in RAW 264.7 macrophages infected with Aux-ZLM vs. Aux-ZRM at 4 hpi and demonstrates the association of DEGs with multiple GO terms. logFC: $\log_2$ fold change.

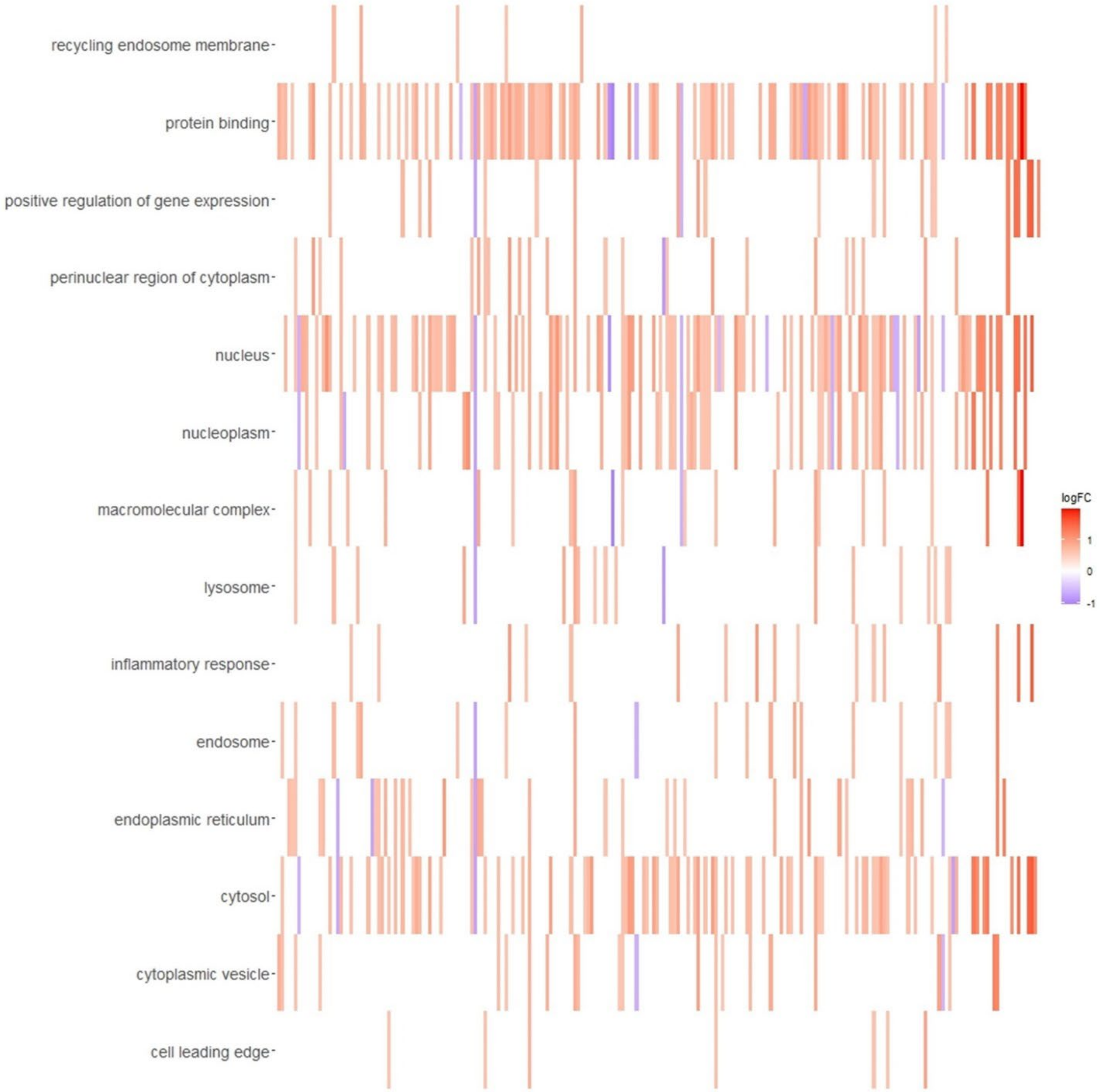

Fig S7: Heatmap of DEGs and all significantly enriched GO terms from RAW 264.7 macrophages infected with Aux-ZLM vs. Aux-ZRM at 4 hpi. Each row is a GO term, and each column is DEG. The $\log_2$ fold changes are shown for the DEGs that are associated with GO terms. DEGs that are not associated with GO terms are assigned the color "white". logFC: $\log_2$ fold change.

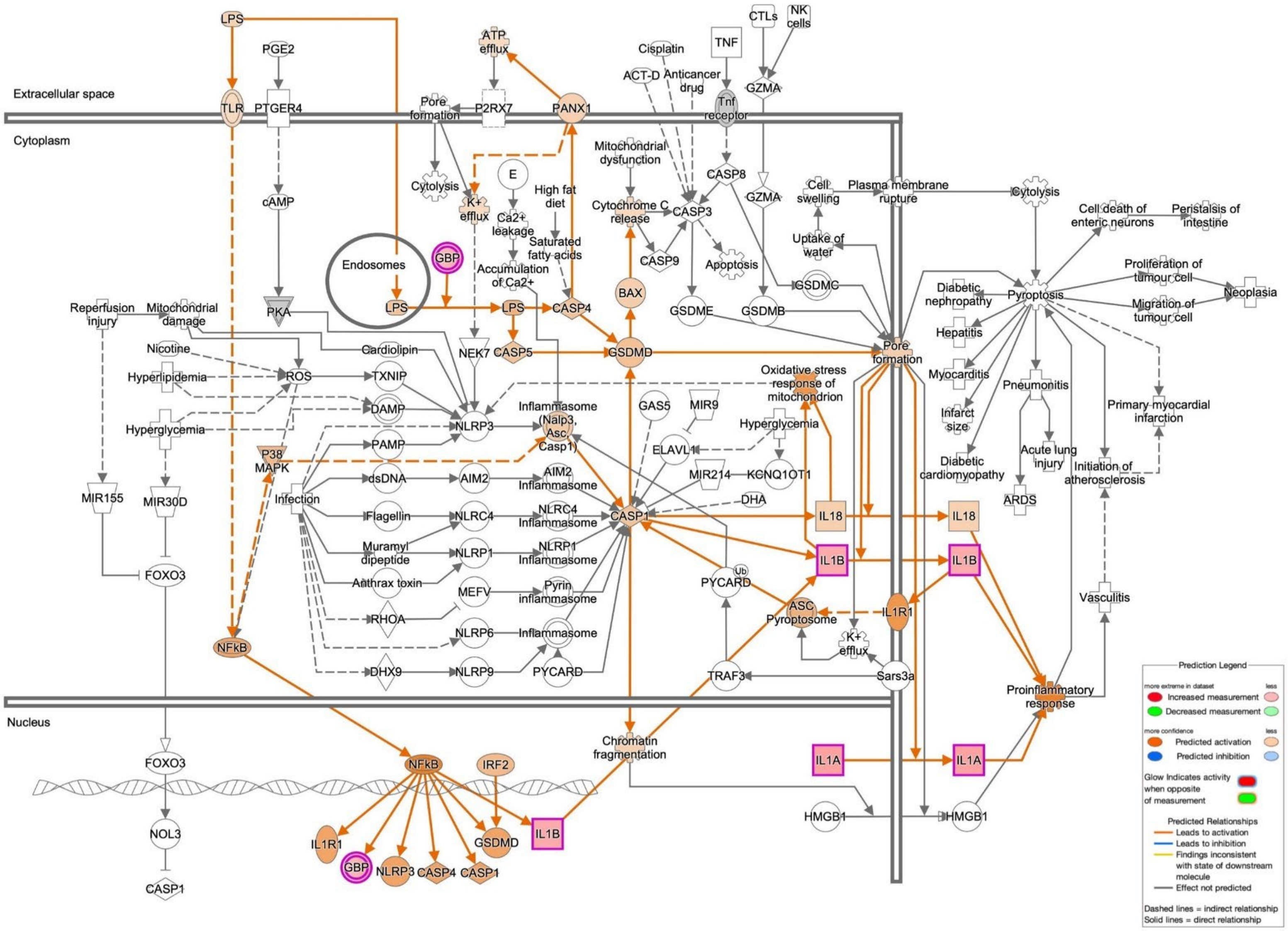

Extracellular space
Cytoplasm
Nucleus
LPS
PGE2
TLR
PTGER4
Pore formation
P2RX7
ATP efflux
PANX1
Cytolysis
K+ efflux
E
Ca2+ leakage
High fat diet
Saturated fatty acids
Accumulation of Ca2+
cAMP
Endosomes
GBP
LPS
CASP4
CASP5
PKA
NEK7
Reperfusion injury
Mitochondrial damage
Nicotine
Hyperlipidemia
Hyperglycemia
ROS
TXNIP
DAMP
Cardiolipin
PAMP
dsDNA
Flagellin
Muramyl dipeptide
Anthrax toxin
RHOA
DHX9
Infection
P38 MAPK
MIR155
MIR30D
FOXO3
NFkB
NLRP3
AIM2
NLRC4
NLRP1
MEFV
NLRP6
NLRP9
Inflammasome (Nalp3, Asc, Casp1)
AIM2 Inflammasome
NLRC4 Inflammasome
NLRP1 Inflammasome
Pyrin inflammasome
Inflammasome
PYCARD
Mitochondrial dysfunction
Cisplatin
ACT-D
Anticancer drug
TNF
Tnf receptor
CTLs
NK cells
GZMA
CASP8
Cytochrome C release
CASP3
CASP9
Apoptosis
BAX
GSDMD
GSDME
GSDMB
GSDMC
Cell swelling
Uptake of water
Plasma membrane rupture
GAS5
MIR9
ELAVL1
Hyperglycemia
MIR214
KCNQ1OT1
DHA
CASP1
Oxidative stress response of mitochondrion
IL18
IL1B
Ub
PYCARD
ASC Pyroptosome
IL1R1
K+ efflux
Sars3a
TRAF3
Chromatin fragmentation
IL1A
HMGB1
Cytolysis
Pyroptosis
Cell death of enteric neurons
Peristalsis of intestine
Proliferation of tumour cell
Migration of tumour cell
Neoplasia
Diabetic nephropathy
Hepatitis
Myocarditis
Infarct size
Diabetic cardiomyopathy
Pneumonitis
Acute lung injury
ARDS
Primary myocardial infarction
Initiation of atherosclerosis
Vasculitis
Proinflammatory response
FOXO3
NOL3
CASP1
IRF2
IL1R1
GBP
NLRP3
CASP4
CASP1
GSDMD
IL1B
Prediction Legend
more extreme in dataset
less
Increased measurement
Decreased measurement
more confidence
less
Predicted activation
Predicted inhibition
Glow Indicates activity when opposite of measurement
Predicted Relationships
Leads to activation
Leads to inhibition
Findings inconsistent with state of downstream molecule
Effect not predicted
Dashed lines = indirect relationship
Solid lines = direct relationship

Fig S8: Predicted activation of the "Pyroptosis Signaling" IPA pathway in RAW 264.7 macrophages infected with Aux-ZLM vs. Aux-ZRM at 24 hpi. The pathway was constructed with IPA.

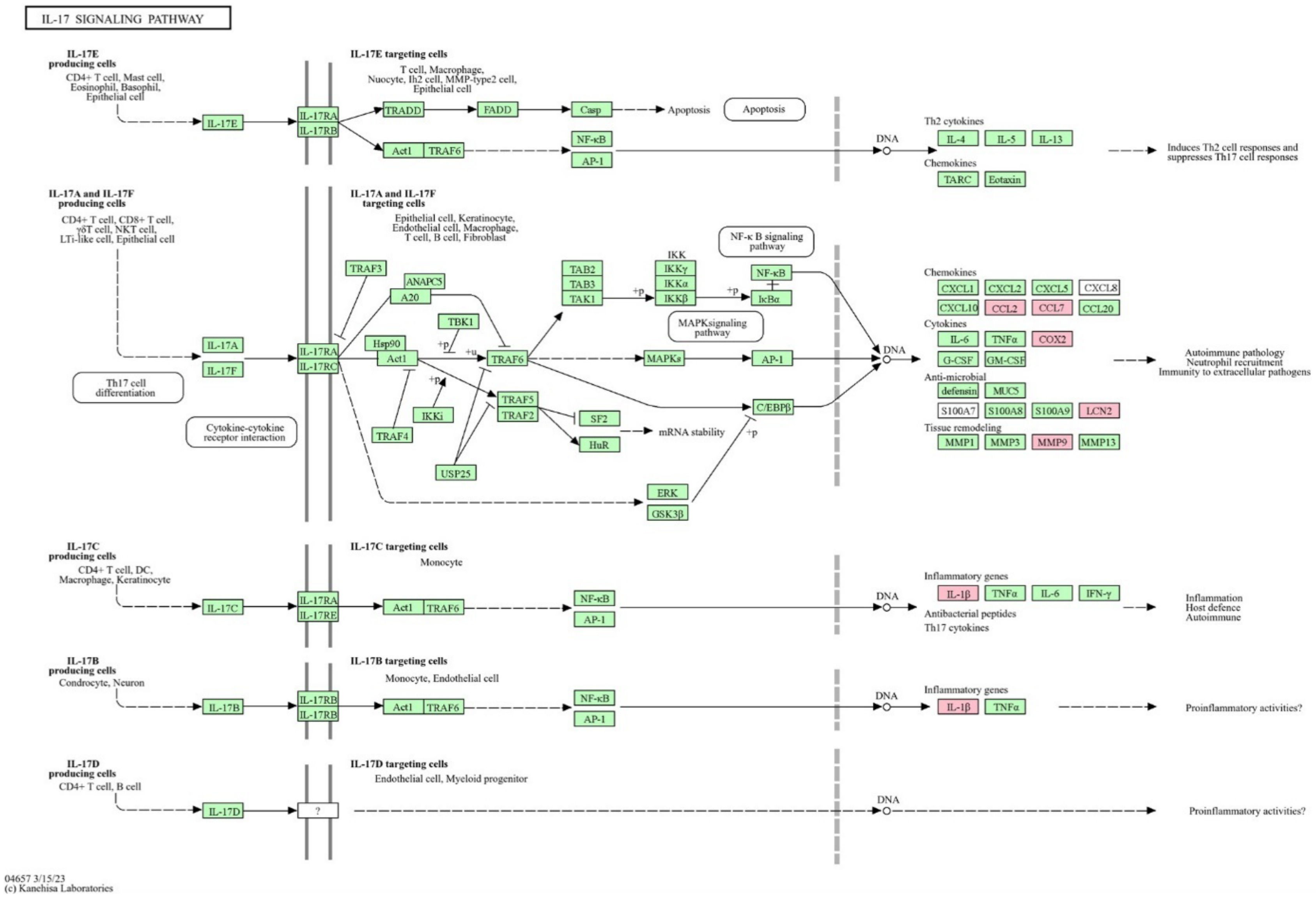
IL-17 SIGNALING PATHWAY
IL-17E producing cells
CD4+ T cell, Mast cell, Eosinophil, Basophil, Epithelial cell
IL-17E
IL-17RA
IL-17RB
IL-17E targeting cells
T cell, Macrophage, Nuocyte, Ih2 cell, MMP-type2 cell, Epithelial cell
TRADD
FADD
Casp
Apoptosis
Apoptosis
Act1
TRAF6
NF-κB
AP-1
DNA
Th2 cytokines
IL-4
IL-5
IL-13
Chemokines
TARC
Eotaxin
Induces Th2 cell responses and suppresses Th17 cell responses
IL-17A and IL-17F producing cells
CD4+ T cell, CD8+ T cell, γδT cell, NKT cell, LTi-like cell, Epithelial cell
IL-17A
IL-17F
Th17 cell differentiation
Cytokine-cytokine receptor interaction
IL-17RA
IL-17RC
IL-17A and IL-17F targeting cells
Epithelial cell, Keratinocyte, Endothelial cell, Macrophage, T cell, B cell, Fibroblast
TRAF3
ANAPC5
A20
Hsp90
Act1
TBK1
+p
+u
TRAF6
+p
IKKi
TRAF4
USP25
TRAF5
TRAF2
SF2
HuR
mRNA stability
TAB2
TAB3
TAK1
+p
IKK
IKKγ
IKKα
IKKβ
+p
NF-κB
IκBα
NF-κ B signaling pathway
MAPKsignaling pathway
MAPKs
AP-1
C/EBPβ
+p
ERK
GSK3β
DNA
Chemokines
CXCL1
CXCL2
CXCL5
CXCL8
CXCL10
CCL2
CCL7
CCL20
Cytokines
IL-6
TNFα
COX2
G-CSF
GM-CSF
Anti-microbial
defensin
MUC5
S100A7
S100A8
S100A9
LCN2
Tissue remodeling
MMP1
MMP3
MMP9
MMP13
Autoimmune pathology
Neutrophil recruitment
Immunity to extracellular pathogens
IL-17C producing cells
CD4+ T cell, DC, Macrophage, Keratinocyte
IL-17C
IL-17RA
IL-17RE
IL-17C targeting cells
Monocyte
Act1
TRAF6
NF-κB
AP-1
DNA
Inflammatory genes
IL-1β
TNFα
IL-6
IFN-γ
Antibacterial peptides
Th17 cytokines
Inflammation
Host defence
Autoimmune
IL-17B producing cells
Condrocyte, Neuron
IL-17B
IL-17RB
IL-17RB
IL-17B targeting cells
Monocyte, Endothelial cell
Act1
TRAF6
NF-κB
AP-1
DNA
Inflammatory genes
IL-1β
TNFα
Proinflammatory activities?
IL-17D producing cells
CD4+ T cell, B cell
IL-17D
?
IL-17D targeting cells
Endothelial cell, Myeloid progenitor
DNA
Proinflammatory activities?
04657 3/15/23
(c) Kanehisa Laboratories

Fig S9: Enrichment of DEGs in the KEGG IL-17 signaling pathway (mmu04657) in RAW 264.7 macrophages infected with Aux-ZLM vs. Aux-ZRM at 24 hpi. Pink genes were upregulated, and green genes were not differentially expressed. The pathway was generated using KEGG Mapper.

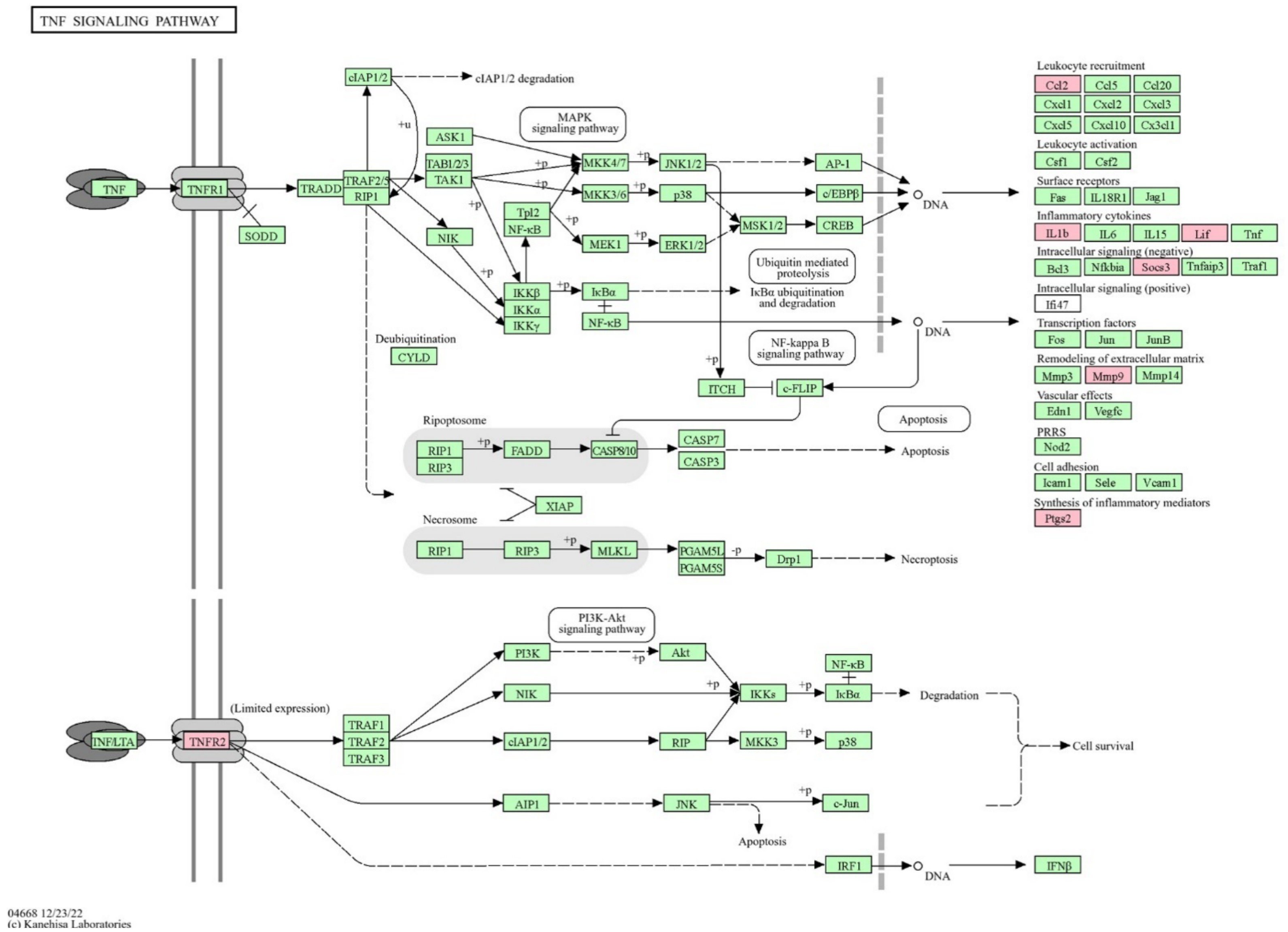

TNF SIGNALING PATHWAY
TNF
TNFR1
SODD
TRADD
TRAF2/5
RIP1
cIAP1/2
cIAP1/2 degradation
+u
ASK1
TAB1/2/3
TAK1
MAPK signaling pathway
MKK4/7
MKK3/6
MEK1
JNK1/2
p38
ERK1/2
Tpl2
NF-κB
NIK
IKKβ
IKKα
IKKγ
IκBα
NF-κB
MSK1/2
AP-1
c/EBPβ
CREB
DNA
Ubiquitin mediated proteolysis
IκBα ubiquitination and degradation
NF-kappa B signaling pathway
ITCH
c-FLIP
Deubiquitination
CYLD
Ripoptosome
RIP1
RIP3
FADD
CASP8/10
CASP7
CASP3
Apoptosis
XIAP
Necrosome
MLKL
PGAM5L
PGAM5S
Drp1
Necroptosis
+p
-p
Leukocyte recruitment
Ccl2
Ccl5
Ccl20
Cxcl1
Cxcl2
Cxcl3
Cxcl5
Cxcl10
Cx3cl1
Leukocyte activation
Csf1
Csf2
Surface receptors
Fas
IL18R1
Jag1
Inflammatory cytokines
IL1b
IL6
IL15
Lif
Tnf
Intracellular signaling (negative)
Bcl3
Nfkbia
Socs3
Tnfaip3
Traf1
Intracellular signaling (positive)
Ifi47
Transcription factors
Fos
Jun
JunB
Remodeling of extracellular matrix
Mmp3
Mmp9
Mmp14
Vascular effects
Edn1
Vegfc
PRRS
Nod2
Cell adhesion
Icam1
Sele
Vcam1
Synthesis of inflammatory mediators
Ptgs2
TNF/LTA
TNFR2
(Limited expression)
TRAF1
TRAF2
TRAF3
PI3K
PI3K-Akt signaling pathway
Akt
NIK
cIAP1/2
RIP
IKKs
MKK3
p38
NF-κB
IκBα
Degradation
Cell survival
AIP1
JNK
c-Jun
Apoptosis
IRF1
DNA
IFNβ
04668 12/23/22
(c) Kanehisa Laboratories

Fig S10: Enrichment of DEGs in the KEGG TNF signaling pathway (mmu04668) in RAW 264.7 macrophages infected with Aux-ZLM vs. Aux-ZRM at 24 hpi. Pink genes were upregulated, and green genes were not differentially expressed. The pathway was generated using KEGG Mapper.

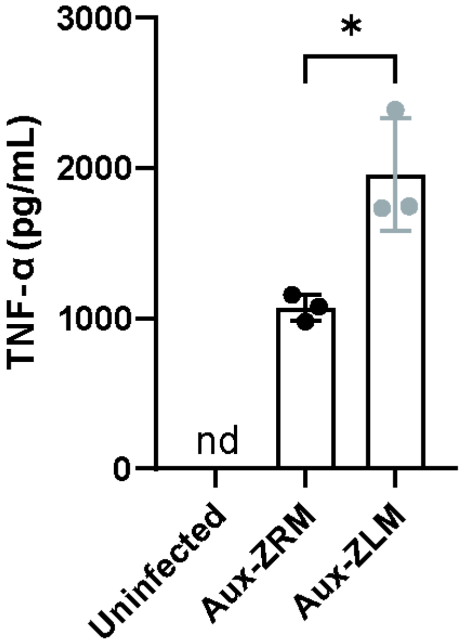

Fig S11: Quantification of TNF-α in RAW 264.7 macrophages. TNF-α was quantified in the cell culture supernatants from either uninfected macrophages or macrophages infected with Aux-ZRM or Aux-ZLM at 4 hpi, as indicated. Note that the later timepoint at 24 hpi could not be compared due to the difference in cell death. There were two and three biological replicates for uninfected and infected conditions, respectively, and each biological replicate was measured in technical duplicate. Standard deviations are shown. Representative results of at least two independent experiments are shown. nd: not detected. *: p-value <0.05.

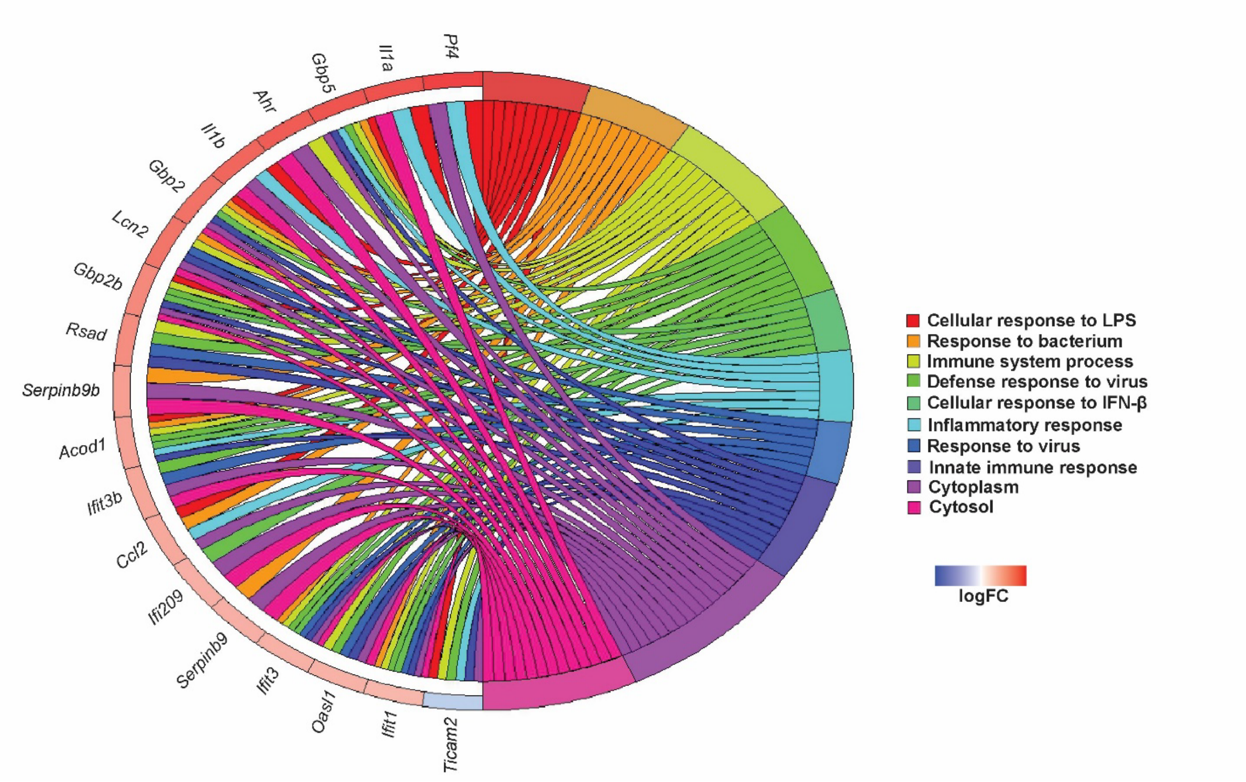

Fig S12: Chord plot of the top 10 GO terms from macrophages infected with Aux-ZLM vs. Aux-ZRM at 24 hpi. The plot was constructed using gene expression in RAW 264.7 macrophages infected with Aux-ZLM vs. Aux-ZRM at 24 hpi and demonstrates the association of DEGs with multiple GO terms. logFC: $\log_2$ fold change.

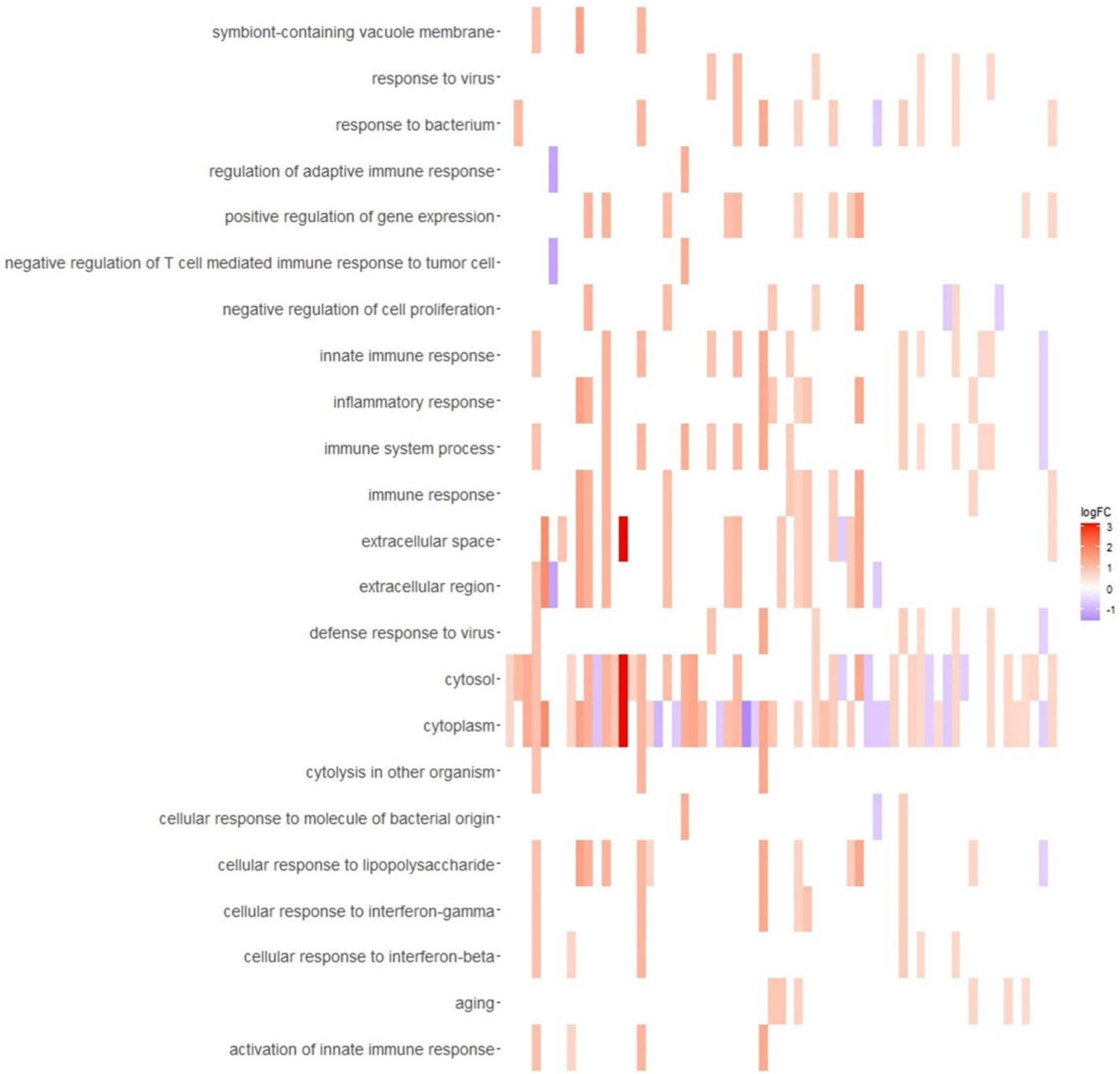

Fig S13: Heatmap of DEGs and all significantly enriched GO terms from RAW 264.7 macrophages infected with Aux-ZLM vs. Aux-ZRM at 24 hpi. Each row is a GO term, and each column is DEG. The $\log_2$ fold changes are shown for the DEGs that are associated with GO terms. DEGs that are not associated with a GO term are assigned the color “white”.

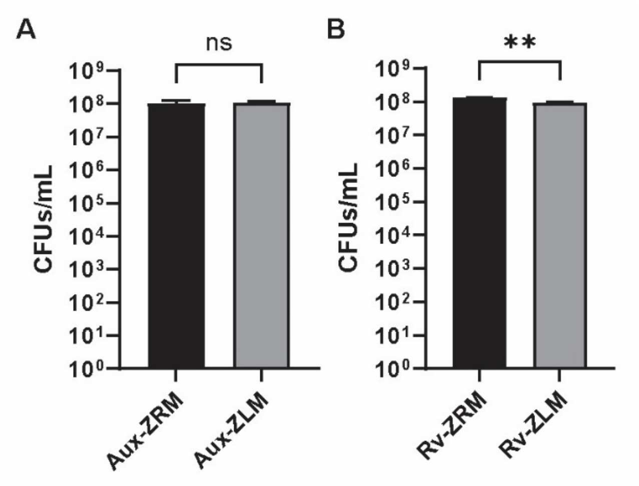

Fig S14: The CFU titers of OD_{600}-adjusted Mtb-Aux and Mtb-Rv. A) Cultures of Aux-ZRM were adjusted to OD_{600} 0.35, Aux-ZLM were adjusted to OD_{600} 0.5. B) Rv-ZRM and Rv-ZLM were adjusted to OD_{600} 0.5. CFU: colony forming unit. ns: not significant. **: p-value <0.01

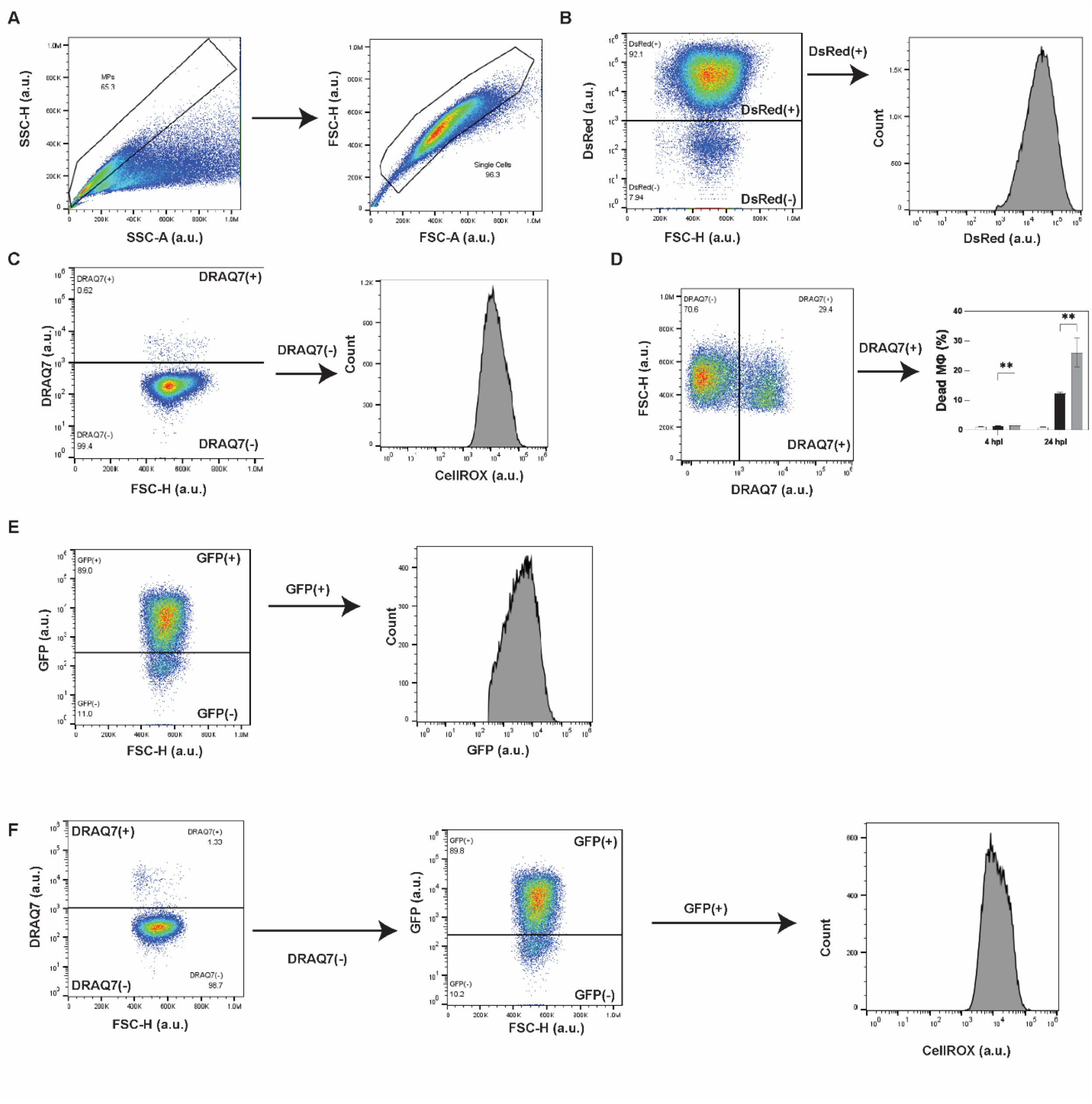

Fig S15: The flow cytometry gating strategies. A) Singlets of RAW 264.7 cells. B) Gating on DsRed-positive macrophages to measure the percentage of infected (DsRed-positive) macrophages and DsRed median fluorescence intensity (MFI). C) Gating on DRAQ7-negative (live) cells to measure ROS production in live macrophages infected with non-fluorescent Mtb-Aux. D) Gating on DRAQ7-positive (dead) cells to measure macrophage death at 4 hpi and 24 hpi. E) Gating on GFP-positive macrophages to measure the percentage of infected (GFP-

positive) macrophages and GFP MFI. F) Gating on DRAQ7-negative GFP-positive macrophages to measure ROS production in macrophages infected with GFP-expressing Mtb-Aux at 4 hpi. FSC-H: forward scatter height. a.u.: arbitrary units. MPs: macrophages. ROS: reactive oxygen species.

SUPPLEMENTARY VIDEOS

Video S1: Confocal microscopy of RAW 264.7 macrophages infected with Aux-ZRM at 24 hpi. Blue: macrophage nuclei. Green: Mtb-Aux. Red: F-actin. Overlayed with brightfield.

Video S2: Confocal microscopy of RAW 264.7 macrophages infected with Aux-ZLM at 24 hpi. Blue: macrophage nuclei. Green: Mtb-Aux. Red: F-actin. Overlayed with brightfield.
